# lncRNA-*PLACT1* sustains activation of NF-κB pathway through a positive feedback loop with IκBα/E2F1 axis in pancreatic cancer

**DOI:** 10.1186/s12943-020-01153-1

**Published:** 2020-02-21

**Authors:** Xiaofan Ren, Changhao Chen, Yuming Luo, Mingyang Liu, Yuting Li, Shangyou Zheng, Huilin Ye, Zhiqiang Fu, Min Li, Zhihua Li, Rufu Chen

**Affiliations:** 1 Department of Medical Oncology, Sun Yat-sen Memorial Hospital, State Key Laboratory of Oncology in South China, 107th Yanjiangxi Road, Yuexiu District, Guangzhou, Guangdong province 510120 People’s Republic of China; 2 Guangdong Provincial Key Laboratory of Malignant Tumor Epigenetics and Gene Regulation, Sun Yat-sen Memorial Hospital, State Key Laboratory of Oncology in South China, 107th Yanjiangxi Road, Yuexiu District, Guangzhou, 510120 Guangdong province People’s Republic of China; 3 Department of Urology, Sun Yat-sen Memorial Hospital, State Key Laboratory of Oncology in South China, 107th Yanjiangxi Road, Yuexiu District, Guangzhou, Guangdong province 510120 People’s Republic of China; 4 Department of Hepatopancreatobiliary Surgery, Sun Yat-sen Memorial Hospital, 107th Yanjiangxi Road, Yuexiu District, Guangzhou, 510120 Guangdong province People’s Republic of China; 5 Department of Medicine, Department of Surgery, the University of Oklahoma Health Sciences Center, 975 NE 10th Street, BRC 1262A, Oklahoma City, OK 73104 USA; 6 Department of General Surgery, Guangdong Provincial People’s Hospital, Guangdong Academy of Medical Sciences, 106th of 2nd Zhongshan Road, Yuexiu District, Guangzhou, Guangdong Province 510080 People’s Republic of China

**Keywords:** Long noncoding RNA, Pancreatic ductal adenocarcinoma, NF-κB signaling pathway, IκBα, Positive feedback loop

## Abstract

**Background:**

The activation of NF-κB signaling pathway is regarded as the dominant process that correlates with tumorigenesis. Recently, increasing evidence shows that long noncoding RNAs (lncRNAs) play crucial roles in sustaining the NF-κB signaling pathway. However, the underlying mechanisms have not yet been elucidated.

**Methods:**

The expression and clinical features of *PLACT1* were analyzed in a 166-case cohort of PDAC by qRT-PCR and in situ hybridization. The functional role of *PLACT1* was evaluated by both in vitro and in vivo experiments. Chromatin isolation by RNA purification assays were utilized to examine the interaction of *PLACT1* with IκBα promoter.

**Results:**

We identified a novel lncRNA-*PLACT1*, which was significantly upregulated in tumor tissues and correlated with progression and poor survival in PDAC patients. Moreover, *PLACT1* promoted the proliferation and invasion of PDAC cells in vitro. Consistently, *PLACT1* overexpression fostered the progression of PDAC both in orthotopic and lung metastasis mice models. Mechanistically, *PLACT1* suppressed IκBα expression by recruiting hnRNPA1 to IκBα promoter, which led to increased H3K27me3 that decreased the transcriptional level of IκBα. Furthermore, E2F1-mediated overexpression of *PLACT1* modulated the progression of PDAC by sustained activation of NF-κB signaling pathway through forming a positive feedback loop with IκBα. Importantly, administration of the NF-κB signaling pathway inhibitor significantly suppressed *PLACT1*-induced sustained activation of NF-κB signaling pathway, leading to reduced tumorigenesis in vivo.

**Conclusions:**

Our findings suggest that *PLACT1* provides a novel epigenetic mechanism involved in constitutive activation of NF-κB signaling pathway and may represent a new therapeutic target of PDAC.

## Background

Pancreatic ductal adenocarcinoma (PDAC) is a devastating digestive system cancer with rapid progression and poor prognosis [1, 2]. Despite various studies of the mechanism and clinical trials, the 5-year survival rate for PDAC remains low at around 9% [3]. One important reason for the dismal prognosis is the highly aggressive nature and early-stage metastasis of PDAC [4, 5]. Therefore, identifying an early diagnostic and therapeutic biomarker involved in PDAC progression is of significant clinical value.

The activation of the nuclear factor κB (NF-κB) signaling pathway is regarded as the dominant process that correlates inflammation with carcinogenesis [6, 7]. When cytokine stimulation acts on the pathway, inhibitory κB (IκB) is phosphorylated by activated IκB kinase (IKK) complex, which induces inhibitory κBα (IκBα) ubiquitination and degradation [8]. Then, NF-κB arrested by IκB in the cytoplasm is released and translocated into the nucleus, resulting in transcriptional activation of various genes [8, 9]. The intensity and duration of NF-κB signaling are regulated by various mechanisms. The oncogene Kras G12D is verified to maintain NF-κB activity by inducing an IL-1a/IKKβ/p62 feedforward loop in PDAC [10]. GSK-3 mediates both classical and non-canonical NF-κB activation and promotes pancreatic cancer cell growth and survival [11, 12]. Nevertheless, the mechanism underlying the constitutive activation of NF-κB signaling pathway in PDAC remains poorly understood.

Long noncoding RNAs (lncRNAs), known as RNAs greater than 200 nt in length and that lack the ability to code for protein, play multiple roles in human cancers through all stages of their development and progression [13, 14]. An increasing number of lncRNAs are characterized by participating in metastasis [15, 16]. Nevertheless, only a fraction of lncRNAs have demonstrated the precise mechanisms for their functions. Several studies revealed that lncRNAs regulate signal transduction in the NF-κB signaling pathway by interacting with the function domain of NF-κB and its transcripts directly [17, 18]. For instance, lncRNA PACER sequesters a repressive subunit of NF-κB in order to enhance the signal [19]. NKILA and Lethe block the activation of the NF-κB signaling pathway through binding to the NF-κB/ IκB complex [20, 21]. Although various lncRNAs have been discovered, the mechanism for their role in regulating the NF-κB signaling pathway is not yet fully elucidated.

In the present study, we reported that a novel lncRNA RP11-1149O23.3, termed pancreatic cancer associated transcript 1 (*PLACT1*), was upregulated in PDAC tissues and was positively associated with poor prognosis of patients with PDAC. *PLACT1* overexpression facilitated PDAC cells proliferation and invasion in vitro and in vivo*.* Moreover, we demonstrated that *PLACT1* downregulated IκBα expression by recruiting heterogeneous nuclear ribonucleoprotein A1 (hnRNPA1) to the IκBα promoter. In addition, E2F transcription factor 1 (E2F1)-mediated overexpression of *PLACT1* modulated the progression of PDAC by sustained activation of the NF-κB signaling pathway through forming a positive feedback loop with IκBα.

## Methods

### Patients and clinical samples

PDAC specimens were obtained from patients who underwent surgery at Sun Yat-sen Memorial Hospital of Sun Yat-sen University between February 2008 and February 2018. Details are provided in Additional file 1.

### RNA pull-down assays

The *PLACT1*-binding proteins were examined using RNA pull-down assays according to the instructions of Magnetic RNA-Protein Pull-Down Kit (Thermo Scientific). Details are provided in Additional file 1.

### Chromatin isolation by RNA purification (ChIRP) assays

The interaction between *PLACT1* and the promoter of IκBα was determined using ChIRP assays according to the instructions of the Magna ChIRP™ Chromatin Isolation by RNA Purification Kit (Millipore, USA). Details are provided in Additional file 1.

### Statistical analysis

All statistical analyses were performed using SPSS 13.0 software (IBM, SPSS, Chicago, IL, USA). Details are provided in Additional file 1.

### Further applied methods

Additional cell culture, lentivirus infection, cell transfection, in situ hybridization (ISH), immunohistochemistry (IHC), qRT-PCR, rapid amplification of cDNA ends (RACE), Cell Counting Kit-8 (CCK-8), EdU, colony formation, wound healing, Transwell, animal treatments, western blotting, RNA Immunoprecipitation (RIP), nuclear-plasma fractionation, immunofluorescence, fluorescence in situ hybridization (FISH), circular dichroism (CD) spectroscopy, fluorescence resonance energy transfer (FRET), dual-luciferase reporter, and Chromatin Immunoprecipitation (ChIP) assays and bioinformatics analysis are further described in the Additional file 1.

## Results

### *PLACT1* was correlated with a poor prognosis in human PDAC

**Fig. 1:**
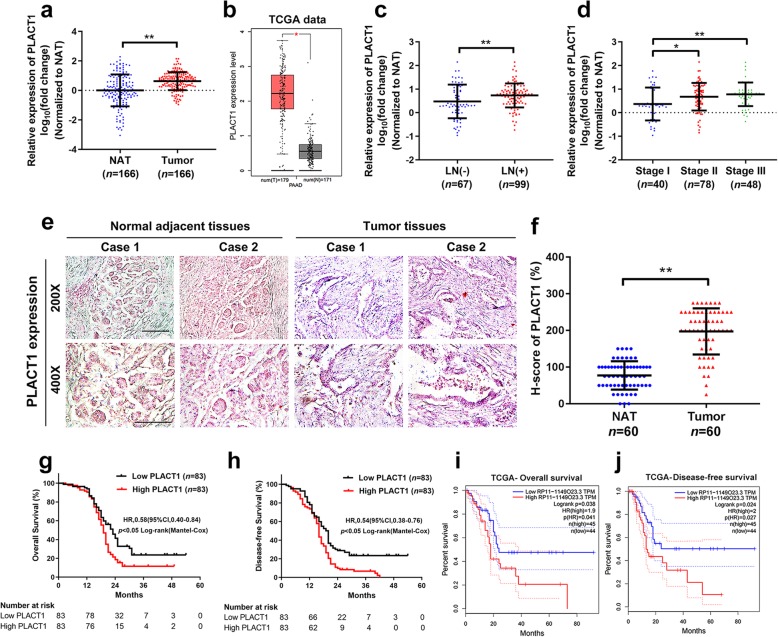
*PLACT1* overexpression is associated with poor prognosis of PDAC. **a** The expression of *PLACT1* in human PDAC tissues (*n* = 166) paired with normal adjacent tissues (*n* = 166) were quantified by qRT-PCR analysis. The results were determined by nonparametric Mann–Whitney U-test. **b** TCGA and Genotype-Tissue Expression (GTEx) data showed that *PLACT1* is upregulated in PDAC tissues (*n* = 179) relative to non-tumorous tissues (*n* = 171). The nonparametric Mann-Whitney U test was used. **c-d** qRT-PCR assays evaluated the correlation of *PLACT1* expression in human PDAC tissues (*n* = 166) with LN status (**c**) and tumor stages (**d**). The results were determined by nonparametric Mann–Whitney U-test. **e-f** ISH analysis of *PLACT1* expression (blue) in the paraffin-embedded NAT (*n* = 60) and tumor sections of PDAC (*n* = 60). Representative images (**e**) from two clinical cases and H-score (**f**) are shown. Statistical significance was assessed by χ^2^ test. Scale bars: 50 μm. **g-h** The Kaplan–Meier curves represented overall survival (**g**) and disease-free survival (**h**) of PDAC patients with low vs. high expression of *PLACT1*. The cutoff value was the median expression of *PLACT1*. **i-j** PDAC patients from the TCGA data were divided into low and high *PLACT1*expression groups; overall survival (**i**) and disease-free survival (**j**) of the patients in the groups used Kaplan-Meier survival analysis. *p*-values was calculated by the log-rank (Mantel-Cox) test. *p < 0.05; **p < 0.01

To identify the critical lncRNAs that involved in PDAC progression, we previously performed microarray analysis on eight PDAC tissues and four non-tumorous tissues (GEO, ID: GSE61166). Twenty-six and Fifty-nine lncRNAs were upregulated and downregulated, respectively, more than 5-fold in PDAC tissues compared with non-tumorous tissues (Additional file 2: Fig. S1a, b). We selected top 5 candidate lncRNAs according to their fold changes for further validation in a larger cohort of 166 cases of PDAC tissues and paired normal adjacent tissues (NAT), as well as in The Cancer Genome Atlas (TCGA) database. We noted that only *PLACT1* was significantly overexpressed in PDAC tissues both in the cohort and TCGA database (*p* < 0.001, Fig. 1a, b). *PLACT1* is located on chromosome 8p21.3 in human and contains a polyadenylated tail at the 3′ terminus (Additional file 2: Fig. S1c, d). The subcellular localization of *PLACT1* was assessed using FISH and subcellular fractionation assays and the results showed that *PLACT1* was localized to both the nuclei and cytoplasm in PDAC cells (Additional file 2: Fig. S1e-g).

Furthermore, analysis of clinical characteristics indicated that *PLACT1* overexpression was closely correlated with lymph node (LN) metastasis and a high pathological tumor stage (Fig. 1c, d and Table 1). Moreover, ISH analysis revealed that a higher *PLACT1* level was detected in the epithelial tissues of PDAC than in normal tissues (Fig. 1e, f). Importantly, Kaplan-Meier analysis demonstrated that patients with high *PLACT1*-expressing PDAC had shorter OS and DFS in our center (Fig. 1g, h) and the TCGA cohorts (Fig. 1i, j), indicating that *PLACT1* upregulation was potentially related to rapid progression of PDAC. The univariate and multivariate Cox regression model revealed that *PLACT1* served as independent factor for poor prognosis in PDAC (Table 2 and Additional file 3: Table S1). Interestingly, *PLACT1* was overexpressed in various human cancers, including gallbladder cancer, colon cancer, rectal cancer, and stomach cancer (Additional file 4: Figure S2a-h), from the TCGA database, and was associated with poor prognosis in mesothelioma and liver hepatocellular cancer (Additional file 4: Figure S2i-l), further supporting the oncogenic role of *PLACT1*. In conclusion, *PLACT1* serves as an important oncogene and is associated with poor clinical outcome of PDAC.

**Table 1:** Correlation between PLACT1 expression and clinicopathologic characteristics of PDAC patients^a^

Characteristics	N of cases	PLACT1 expression level		
		High	Low	*p*-value
Total cases	166	83	83	
Age				0.425
≤ 60	64	29	35	
> 60	102	54	48	
Gender				0.532
Male	93	44	49	
Female	73	39	34	
Differentiation				0.788
Well	29	15	14	
Moderately	106	51	55	
Poor	31	17	14	
T stage				0.164
T1	50	22	28	
T2	48	20	28	
T3	57	35	22	
T4	11	6	5	
TNM stage (AJCC) ^b^				0.008^**^
Stage I	40	12	28	
Stage II	78	41	37	
Stage III	48	30	18	
Lymphatic metastasis				0.011^*^
Negative	67	25	42	
Positive	99	58	41	

Abbreviations: *N of cases* number of cases, *T stage* tumor stage, *TNM* tumor node metastasis.

^a^ Chi-square test, ^*^p < 0.05, ^**^p < 0.01

^b^ American Joint Committee on Cancer (AJCC), patients were staged in accordance with the 8th Edition of the AJCC Cancer’s TNM Classification

**Table 2:** Univariate and multivariate analysis of Overall Survival (OS) in PDAC patients (*n* = 166)

Variables	Univariate analysis			Multivariate analysis		
	HR	95%CI	*p*-Value	HR	95%CI	*p*-Value
Age	0.702	0.485–1.017	0.062			
Gender	0.825	0.582–1.171	0.283			
Differentiation (moderately or poor vs. well)	1.245	0.772–2.008	0.369			
T stage (T3 or T4 vs. T1 or T2)	1.396	0.981–1.986	0.064			
TNM stage (AJCC) (stage II or stage III vs. stage I)	3.250	2.003–5.274	0.001^**^	1.888	1.001–3.559	0.050
Lymphatic metastasis	2.842	1.928–4.189	0.001^**^	1.900	1.149–3.140	0.012^*^
PLACT1 expression	1.645	1.154–2.345	0.006^**^	1.460	1.017–2.096	0.040^*^

Abbreviations: *HR* hazard ratio, *95%CI* 95% confidence interval, *T stage* tumor stage, *TNM* tumor node metastasis. Cox regression analysis, ^*^p < 0.05, ^**^p < 0.01

### *PLACT1* promotes proliferation, migration, and invasion of PDAC cells

**Fig. 2:**
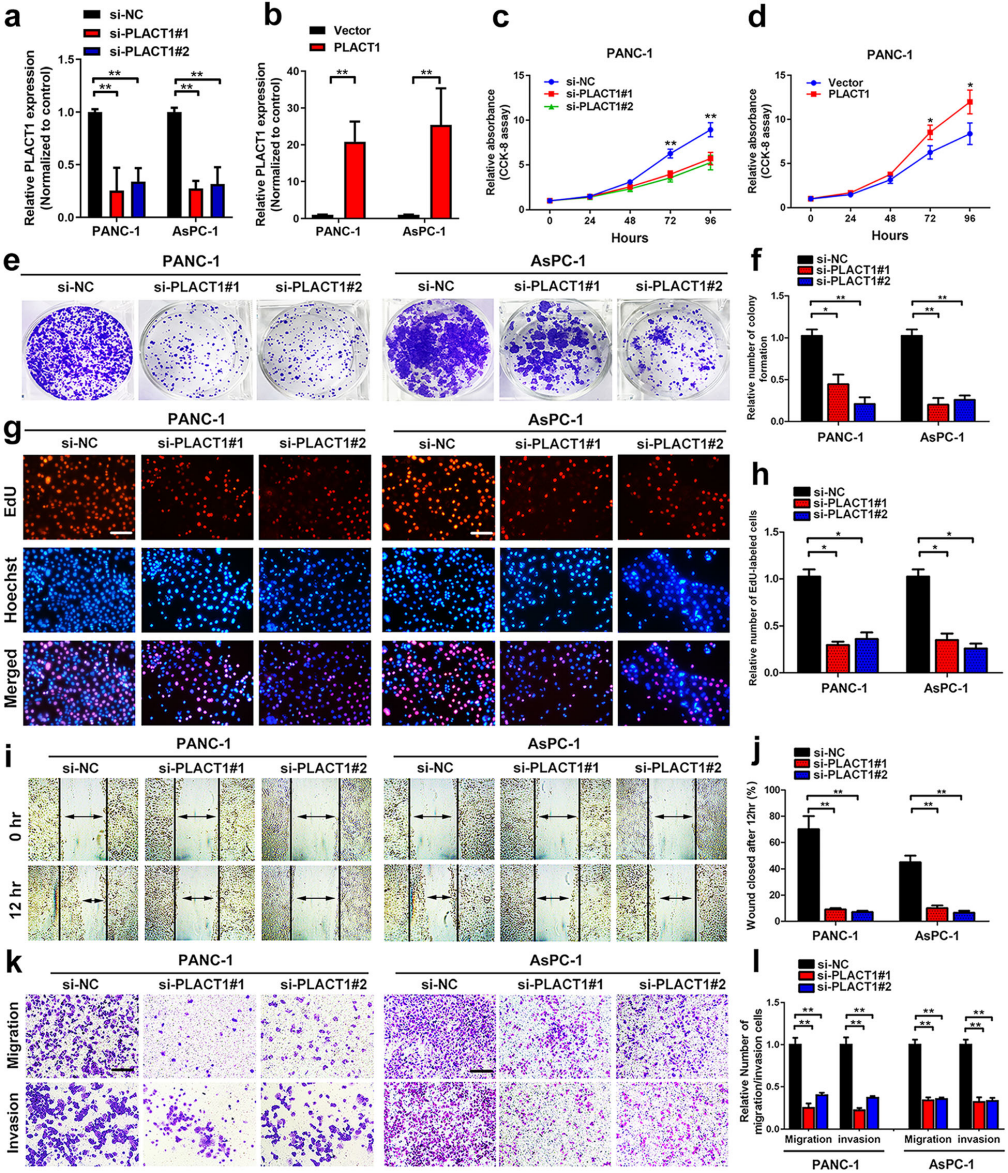
*PLACT1* promotes proliferation, migration, and invasion of PDAC cells. a-b, Efficiencies of *PLACT1* knockdown (**a**) and overexpression (**b**) in PANC-1 and AsPC-1 cells were verified by qRT-PCR assays. **c-d** The cell viability of si-*PLACT1*-transfected (**c**) or *PLACT1*-cDNA-transfected (**d**) PANC-1 cells by CCK-8 assays. **e-f** Effect of *PLACT1* knockdown on colony formation was counted in PANC-1 and AsPC-1 cells (**e**). The histogram analysis (f) showed the mean ± *SD* of colonies from three independent experiments. **g-h** Representative images (**g**) and histogram analysis (**h**) of EdU assays after *PLACT1* knockdown in PANC-1 and AsPC-1 cells. **i-j** Representative images (**i**) and histogram analysis (**j**) of wound healing assays after *PLACT1* knockdown in PANC-1 and AsPC-1 cells. **k-l** Representative images (**k**) and histogram analysis (**l**) of Transwell assays after *PLACT1* knockdown in PANC-1 and AsPC-1 cells. Scale bars: 100 μm. Significance level was assessed using two-tailed *t*-tests and one-way analysis of variance (ANOVA) followed by Dunnett’s tests for multiple comparison. Figures with error bars show standard deviations of three independent experiments. *p < 0.05 and **p < 0.01

Considering *PLACT1* overexpression in PDAC, we further investigated whether it contributed to PDAC progression. First, we analyzed the expression of *PLACT1* in seven PDAC cell lines (AsPC-1, BxPC-3, Capan-2, CFPAC-1, MIA PaCa-2, PANC-1 and SW1990) and normal pancreatic cell line HPNE. The results showed that the highest expression of *PLACT1* was found in both PANC-1 and AsPC-1 (Additional file 5: Figure S3a). Thus, we chose these PDAC cell lines for further investigation. PDAC cells were transfected with small interfering RNAs (siRNAs) targeted to *PLACT1* and *PLACT1*-pcDNA3.1 vector separately, which sufficiently reduced or increased the expression of *PLACT1* (Fig. 2a, b). CCK-8 assays showed that the viability of PANC-1 and AsPC-1 decreased significantly after downregulating *PLACT1* (Fig. 2c and Additional file 5: Figure S3b), whereas overexpression of *PLACT1* increased cell viability in both PANC-1 and AsPC-1 cells (Fig. 2d and Additional file 5: Figure S3c). Colony formation assays revealed that *PLACT1* knockdown significantly reduced cell colonies compared with NC (Fig. 2e, f), whereas *PLACT1* overexpression had the opposite effect (Additional file 5: Figure S3d). Moreover, downregulating *PLACT1* expression led to a significant inhibition in proliferation conducted by EdU assays in PDAC cells (Fig. 2g, h). Meanwhile, *PLACT1* overexpression obviously promoted proliferation in PANC-1 and AsPC-1 cells (Additional file 5: Figure S3e). These results indicate that *PLACT1* participates in proliferation of PDAC cells in vitro.

Furthermore, we found that *PLACT1* overexpression promotes the migration and invasion of PDAC cells. Wound healing assays showed that *PLACT1* knockdown remarkably suppressed PDAC cell mobility (Fig. 2i, j), whereas *PLACT1* overexpression had the opposite effect (Additional file 5: Figure S3f). The results of Transwell assays were similar to those of wound healing assays (Fig. 2k, l and Additional file 5: Figure S3g). Together, these findings show that *PLACT1* overexpression facilitates migration and invasion of PDAC cells in vitro.

KRAS or p53 mutation are frequent oncogenic events observed in PDAC. To exclude the possibility that *PLACT1* promoted proliferation, migration, and invasion of PDAC cells in a KRAS/p53 mutation-dependent manner, we further analyzed the functions of *PLACT1* in BxPC-3 (KRAS wild-type cell line) and Capan-2 (p53 wild-type cell line). We found that *PLACT1* overexpression facilitated the proliferation, migration, and invasion abilities in BxPC-3 and Capan-2, while *PLACT1* knockdown significantly inhibited the proliferation, migration, and invasion of BxPC-3 and Capan-2, suggesting that *PLACT1* promotes proliferation, migration, and invasion of PDAC independent of KRAS/p53 (Additional file 5: Figure S3h, i and Additional file 6: Figure S4a-h).

### *PLACT1* promotes PDAC tumorigenicity and metastatic potential in vivo

**Fig. 3:**
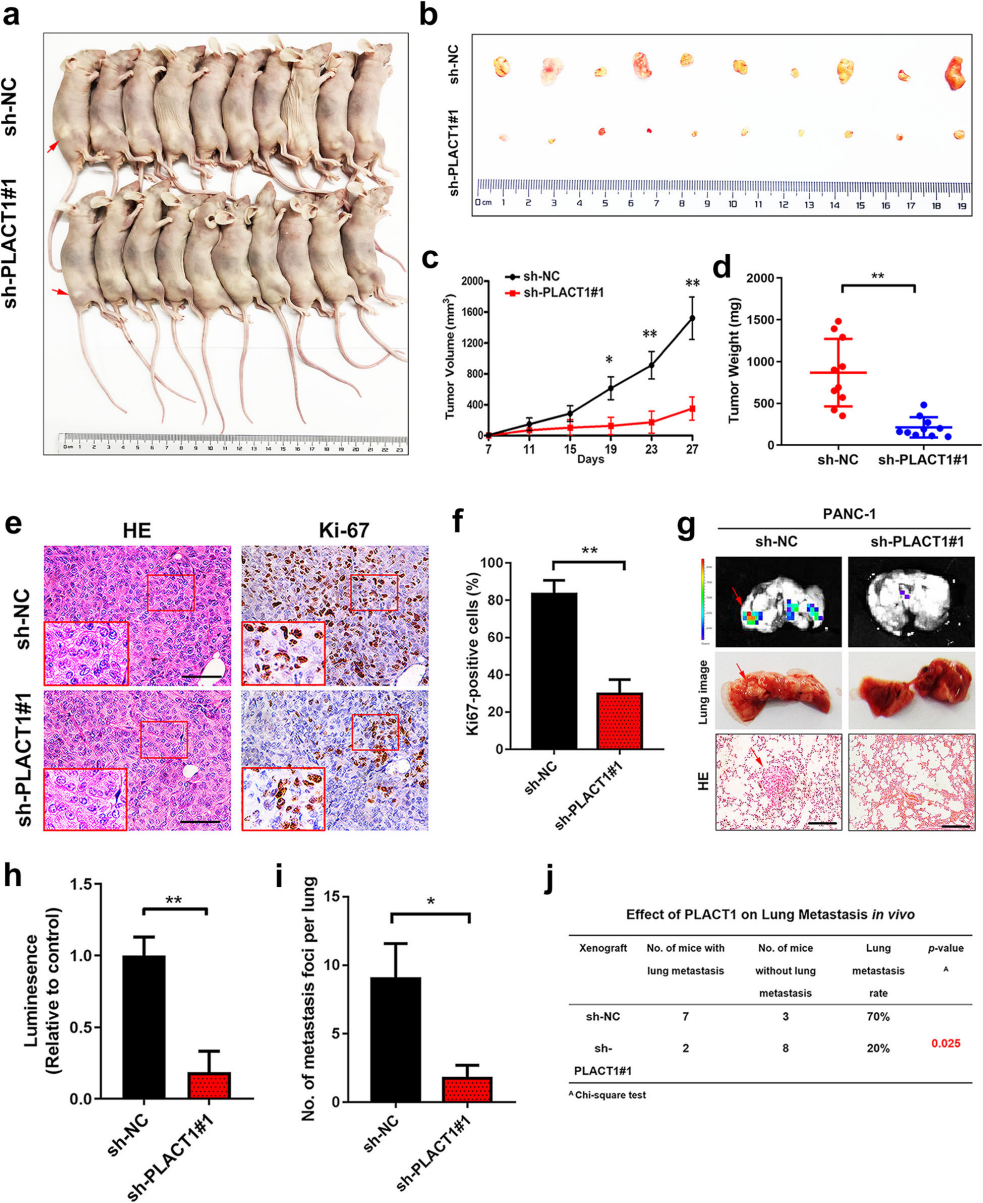
*PLACT1* overexpression promotes a significant effect on tumorigenesis and metastatic potential in vivo. **a-b** Gross appearance of xenograft tumors after subcutaneous injections with sh-NC and sh-*PLACT1*#1 group (*n* = 10). **c-d** Tumor volumes (**c**) and weights (**d**) were measured in the indicated groups (*n* = 10). **e-f** Representative images (**e**) of IHC for Ki-67. Histogram analysis (**f**) revealed that *PLACT1* was associated with Ki-67 expression (*n* = 10). Scale bars: 50 μm. **g** Representative images of lung colonization after injection of PANC-1 cells into the tail veins of mice (*n* = 10). Scale bars: 50 μm. **h-i** Histogram analysis for luminescence (**h**) and the number (**i**) of metastatic foci representing lung metastasis (*n* = 10). **j** The ratio of lung metastasis was calculated for indicated group (*n* = 10). Statistical significance was assessed using two-tailed *t*-tests and ANOVA followed by Dunnett’s tests for multiple comparison. Error bars represent standard deviations of three independent experiments. *p < 0.05 and **p < 0.01

To further evaluate the oncogenic role of *PLACT1* on PDAC cells in vivo, we established xenograft mouse models (*n* = 10 per group). The results showed that *PLACT1* knockdown by stable transfection with sh-*PLACT1*#1 suppressed tumor growth (Fig. 3a, b). Compared with the sh-NC group, a significant decrease in tumor size and tumor weight was observed in the sh-*PLACT1*#1 group (Fig. 3c, d). In addition, IHC assays showed that a lower level of Ki-67 was detected in the cancer tissues of *PLACT1*-silencing mice (Fig. 3e, f).

We further explored the potential effect of *PLACT1* on PDAC metastasis in a tail vein injection model (*n* = 10 per group). Consistently, fewer pulmonary metastatic foci and lower metastatic rate were present in the sh-*PLACT1*#1 group than in the NC group (Fig. 3g-j). The results showed that silencing *PLACT1* significantly impaired the metastasis of tumor cells to the lung, indicating that *PLACT1* promoted the metastasis of PDAC.

**Fig. 4:**
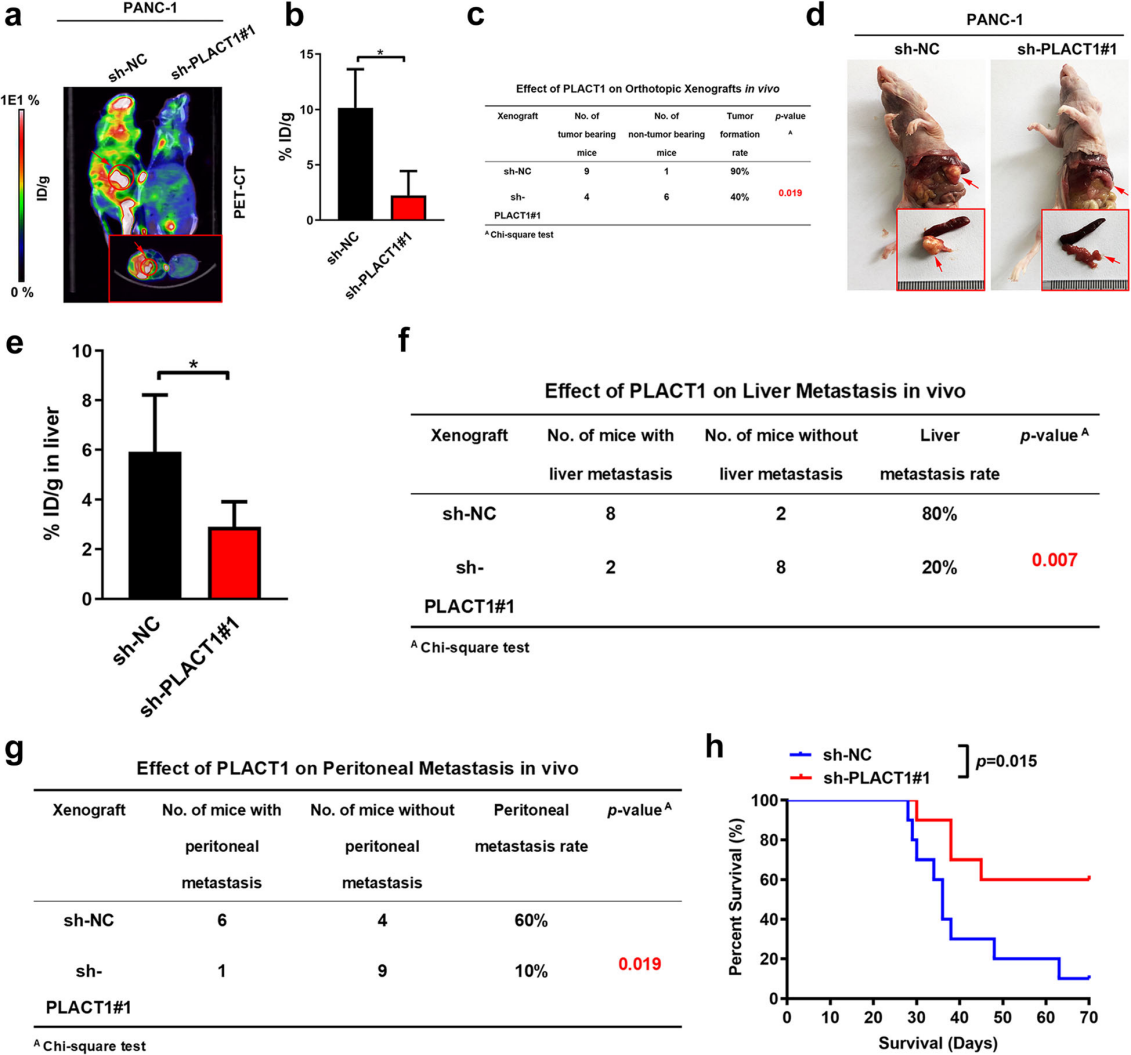
*PLACT1* facilitates the tumorigenesis and metastasis of PDAC in an orthotopic xenograft model. **a-b** Representative PET-CT images (**a**) and histogram analysis (**b**) of ^18^FDG accumulation in pancreas in orthotopic xenografts after orthotopically injections with indicated PANC-1 cells (*n* = 10). The ^18^FDG concentrations in orthotopic tumor were normalized to %ID/g. **c** The tumor formation rate of orthotopic xenograft was calculated for indicated group (*n* = 10). **d** Representative images of orthotopic tumors after orthotopically injections with indicated PANC-1 cells. **e** Histogram analysis of ^18^FDG accumulation in liver in orthotopic xenografts after orthotopically injections with indicated PANC-1 cells (*n* = 10). **f-g** The liver metastasis (**f**) and peritoneal metastasis (**g**) rate of orthotopic xenograft was calculated for indicated group (*n* = 10). **h** Survival analysis for orthotopic tumor bearing mice in indicated group (*n* = 10). Statistical significance was assessed using two-tailed *t*-tests and ANOVA followed by Dunnett’s tests for multiple comparison. Error bars represent standard deviations of three independent experiments. *p < 0.05 and **p < 0.01

Given that orthotopic xenograft models were considered more clinically relevant to simulating the anatomy and physiology of PDAC, we further explored the role of *PLACT1* on tumorigenesis and metastasis by orthotopic transplantation of PDAC cells (*n* = 10 per group). Positron emission tomography and computed tomography (PET-CT) scanning showed that ^18^F-fluorodeoxyglucose (^18^FDG) accumulation in pancreas was critically reduced in mice bearing *PLACT1*- silencing cells, indicating that *PLACT1* knockdown inhibited tumor growth (Fig. 4a, b). Intriguingly, *PLACT1*-silencing caused a lower cancer incidence in mice received orthotopic inoculation of PDAC cells (Fig. 4c). Moreover, decreased tumor size was obtained in the sh-*PLACT1*#1 group compared with the sh-NC group (Fig. 4d). Furthermore, lower ^18^FDG accumulation in liver was observed in sh-*PLACT1* group compared with the control (Fig. 4e). *PLACT1*-silencing reduced the incidence of peritoneal and liver metastasis in tumor-bearing mice, suggesting that *PLACT1* promoted metastasis of PDAC (Fig. 4f, g). Additionally, survival analysis showed that *PLACT1*-silencing prolonged the survival time of mice compared with the control group (Fig. 4h). Taken together, these results indicate that *PLACT1* enhanced PDAC progression both in vitro and in vivo.

### *PLACT1* directly binds to hnRNPA1

**Fig. 5:**
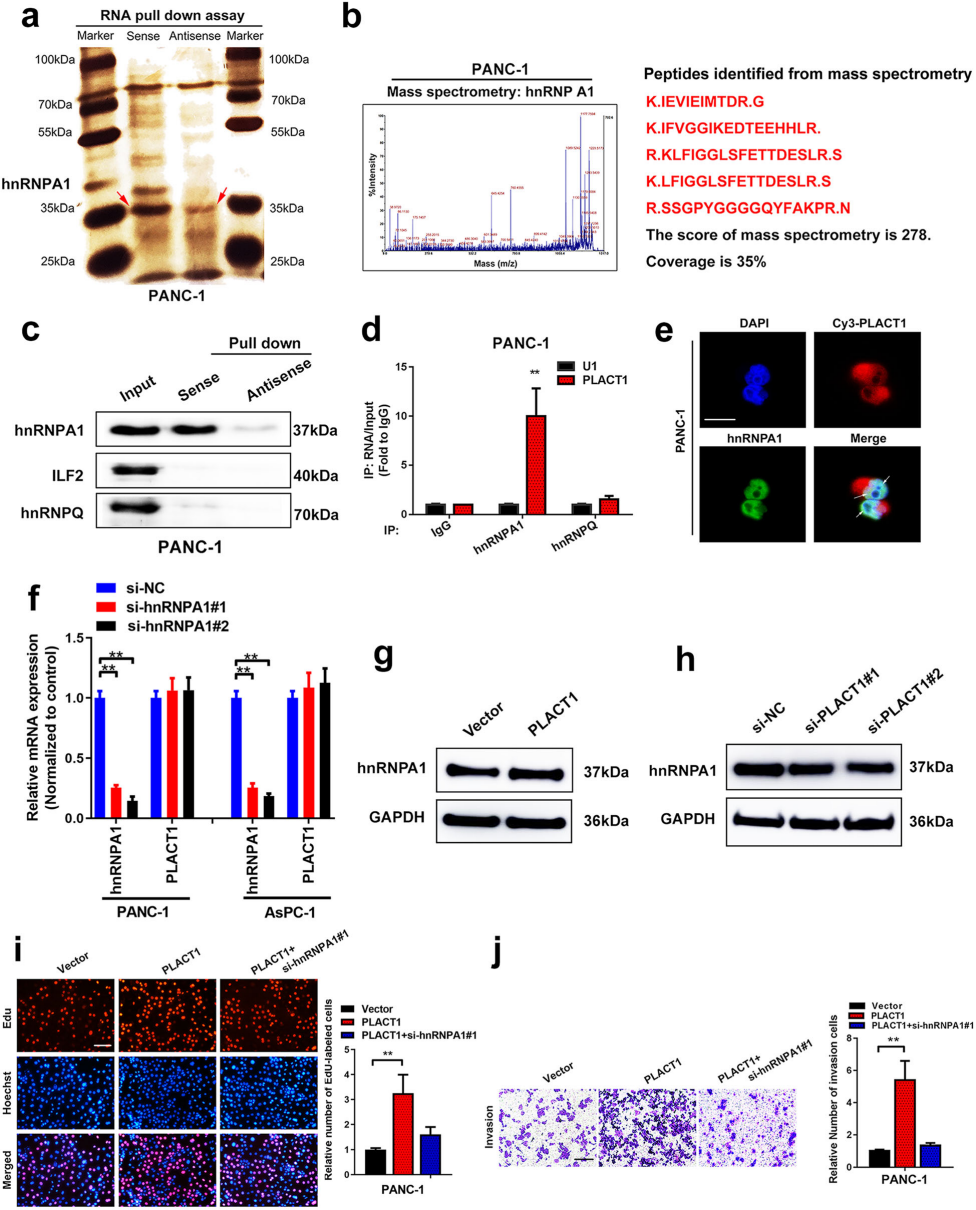
*PLACT1* directly interacts with hnRNPA1. **a***PLACT1* sense and antisense RNAs were used in pull-down assays in PANC-1 cells, followed by electrophoresis and silver staining. HnRNPA1 is shown by a red arrow. **b** Mass spectrometry assays identified the *PLACT1*-interacting protein as hnRNPA1. **c** Western blotting analysis of proteins captured by *PLACT1* sense and antisense fragments, indicating that *PLACT1* associates with hnRNPA1. **d** RIP assays revealed that *PLACT1* bound to hnRNPA1. **e** The colocalization of *PLACT1* and hnRNPA1 was assessed by FISH and immunofluorescence. Scale bar: 5 μm. **f** qRT-PCR analysis indicated efficiency of hnRNPA1 knockdown and *PLACT1* expressions in the hnRNPA1 knockdown cells. **g-h** Western blotting analysis showed the hnRNPA1 expression after *PLACT1* overexpression (g) or knockdown (h) in PDAC. **i-j** EdU (i) and Transwell (j) assays revealed that depletion of hnRNPA1 partly reversed the effects of *PLACT1*-overexpressing PANC-1 cells. Representative images (left panel) and histogram analysis (right panel) are shown. Scale bars: 100 μm. *p-*values were calculated by using two-tailed *t*-tests and ANOVA followed by Dunnett’s tests for multiple comparison. The error bars represent standard deviations of three independent experiments. *p < 0.05 and **p < 0.01

LncRNAs have been reported to exert biological functions by interacting with proteins [22]. Therefore, to confirm *PLACT1*-binding proteins, RNA pull-down assays were performed by using biotin-labeled *PLACT1* and antisense control in PANC-1 cells (Fig. 5a). Subsequent silver staining showed a distinct band weighted between 30 and 45 kDa, which was identified as hnRNPA1 by mass spectrometry (MS) (Fig. 5b). Moreover, western blotting analysis indicated that *PLACT1* was associated with hnRNPA1, as indicated by the pull-down assays (Fig. 5c). Whether this candidate protein can directly interact with *PLACT1* was evaluated by RIP assays. The results showed significant interaction of *PLACT1* with hnRNPA1 in PANC-1 and AsPC-1 cells (Fig. 5d and Additional file 7: Figure S5a). Furthermore, FISH and immunostaining showed that *PLACT1* and hnRNPA1 were co-localized in PANC-1 cells (Fig. 5e). However, hnRNPA1 depletion did not affect the expression levels of *PLACT1* (Fig. 5f), while overexpression and knockdown of *PLACT1* did not influence the expression levels of hnRNPA1(Fig. 5g, h and Additional file 7: Figure S5b, c), suggesting that there was no mutual regulatory relationship between *PLACT1* and hnRNPA1.

As hnRNPA1 contributes to the progression of multiple cancers, we further explored whether hnRNPA1 acted as an oncogene in PDAC. We found that hnRNPA1 was upregulated in PDAC tissues compared with NATs (Additional file 7: Figure S5d, e). Consistently, analysis of TCGA dataset confirmed that hnRNPA1 was overexpressed in PDAC (Additional file 7: Figure S5f). Moreover, hnRNPA1 overexpression correlated with shorter overall survival of PDAC patients (Additional file 7: Figure S5g). We performed rescue experiments to determine whether the interaction between hnRNPA1 and *PLACT1* contributed to PDAC progression. We found that *PLACT1* overexpression could promote the increase in PDAC cell proliferation and invasion, and knockdown of hnRNPA1 was able to partly reverse these effects (Fig. 5i, j and Additional file 7: Figure S5h-o). These results further confirm the interaction between hnRNPA1 and *PLACT1*, and that *PLACT1* plays a crucial role in PDAC progression.

### *PLACT1* induces activation of the NF-κB signaling pathway in an IκBα-dependent manner

**Fig. 6:**
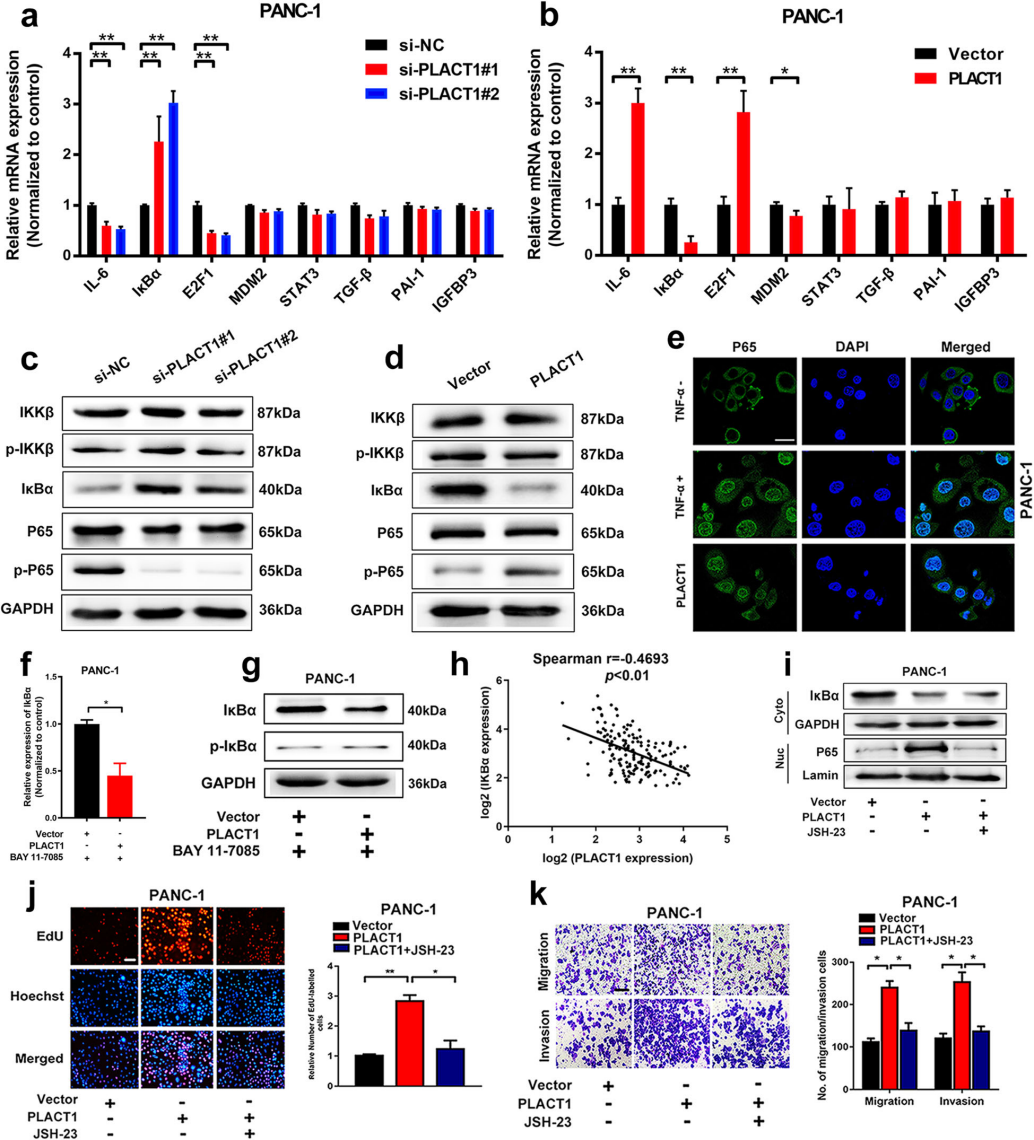
*PLACT1* activates the NF-κB signaling pathway. **a-b** Genes involved in the NF-κB signaling pathway were detected by qRT-PCR in *PLACT1* knockdown (**a**) or overexpression (**b**) cells. **c-d** Western blotting analysis showed the changed protein levels involved in the NF-κB signaling pathway after knockdown (**c**) or overexpression (**d**) of *PLACT1* in PANC-1 cells. **e** Representative images of immunofluorescence analysis showed that *PLACT1* induced P65 translocation in PANC-1. **f-g** qRT-PCR (**f**) and western blotting (**g**) analysis indicated that *PLACT1* downregulated the mRNA and protein levels of IκBα in PANC-1 after treatment with BAY 11–7085. **h***PLACT1* was negatively correlated with IκBα in 166-case of PDAC tissues. **i**, Western blotting analysis showed that *PLACT1*-mediated P65 translocation can be reversed by JSH-23. **j-k** Representative images and histogram analysis of EdU (**j**) and Transwell (**k**) assays showed that JSH-23 reversed the effects of *PLACT1*-overexpressing PANC-1 cells. Scale bars: 100 μm. *p-*values were calculated by using two-tailed *t*-tests and ANOVA followed by Dunnett’s tests for multiple comparison. The error bars represent standard deviations of three independent experiments. *p < 0.05 and **p < 0.01

Previous studies showed that hnRNPA1 contributed to the activation of the NF-κB signaling pathway [23]. It could be inferred that hnRNPA1 played an important role in the activation of the NF-κB signaling pathway as a nucleocytoplasmic shuttling protein. Therefore, we performed qRT-PCR and western blotting assays to assess changed genes involved in the NF-κB signaling pathway in PDAC cells. The results showed that IκBα expression was downregulated by *PLACT1* overexpression, whereas it was upregulated by *PLACT1* knockdown in PANC-1 and AsPC-1 cells (Fig. 6a-d and Additional file 8: Figure S6a, b). However, neither overexpression nor downregulation of *PLACT1* affected the phosphorylation of IKK in PANC-1 cells (Fig. 6c, d), suggesting that *PLACT1* modulated the NF-κB signaling pathway by influencing IκBα expression rather than IKK activation. Moreover, immunofluorescence assays showed that the translocation of P65 into the nucleus was dramatically enhanced in PDAC cells by ectopic *PLACT1* expression, confirming that *PLACT1* induced the NF-κB signaling pathway activation in PDAC (Fig. 6e).

Previous studies reported that both IκBα phosphorylation and decreased IκBα transcription level could lead to IκBα degradation [24, 25]. In the present study, BAY 11–7085, which is recognized as the inhibitor of IκBα phosphorylation, was added to *PLACT1*-overexpressing PDAC cells or NC cells to assess the expression of IκBα. Interestingly, we found that the IκBα expression was downregulated in *PLACT1*-expressing plasmid-treated PDAC compared with cells treated with the corresponding empty vectors after treatment with BAY 11–7085 (Fig. 6f, g and Additional file 8: Figure S6c), suggesting that *PLACT1*-mediated IκBα expression occurred primarily through transcriptional regulation. In addition, we found a negative correlation between *PLACT1* and IκBα mRNA level in 166-case of PDAC tissues, which further confirmed that *PLACT1* regulated IκBα at transcriptional level rather than post-translational level (Fig. 6h).

We further evaluated whether *PLACT1* affected the progression of PDAC via activation of the NF-κB signaling pathway. We found that *PLACT1* overexpression enhanced the activation of the NF-κB signaling pathway and treatment with NF-κB inhibitor, JSH-23, significantly suppressed *PLACT1*-induced NF-κB signaling pathway activation (Fig. 6i). Moreover, inhibition of the NF-κB signaling pathway with JSH-23 partly impaired *PLACT1*-induced proliferation and metastasis of PDAC cells (Fig. 6j, k and Additional file 8: Figure S6d-i). Taken together, these data suggest that *PLACT1* activates the NF-κB signaling pathway to facilitate the progression of PDAC in an IκBα-dependent manner.

### *PLACT1* forms triplexes with the promoter sequences of IκBα

**Fig. 7:**
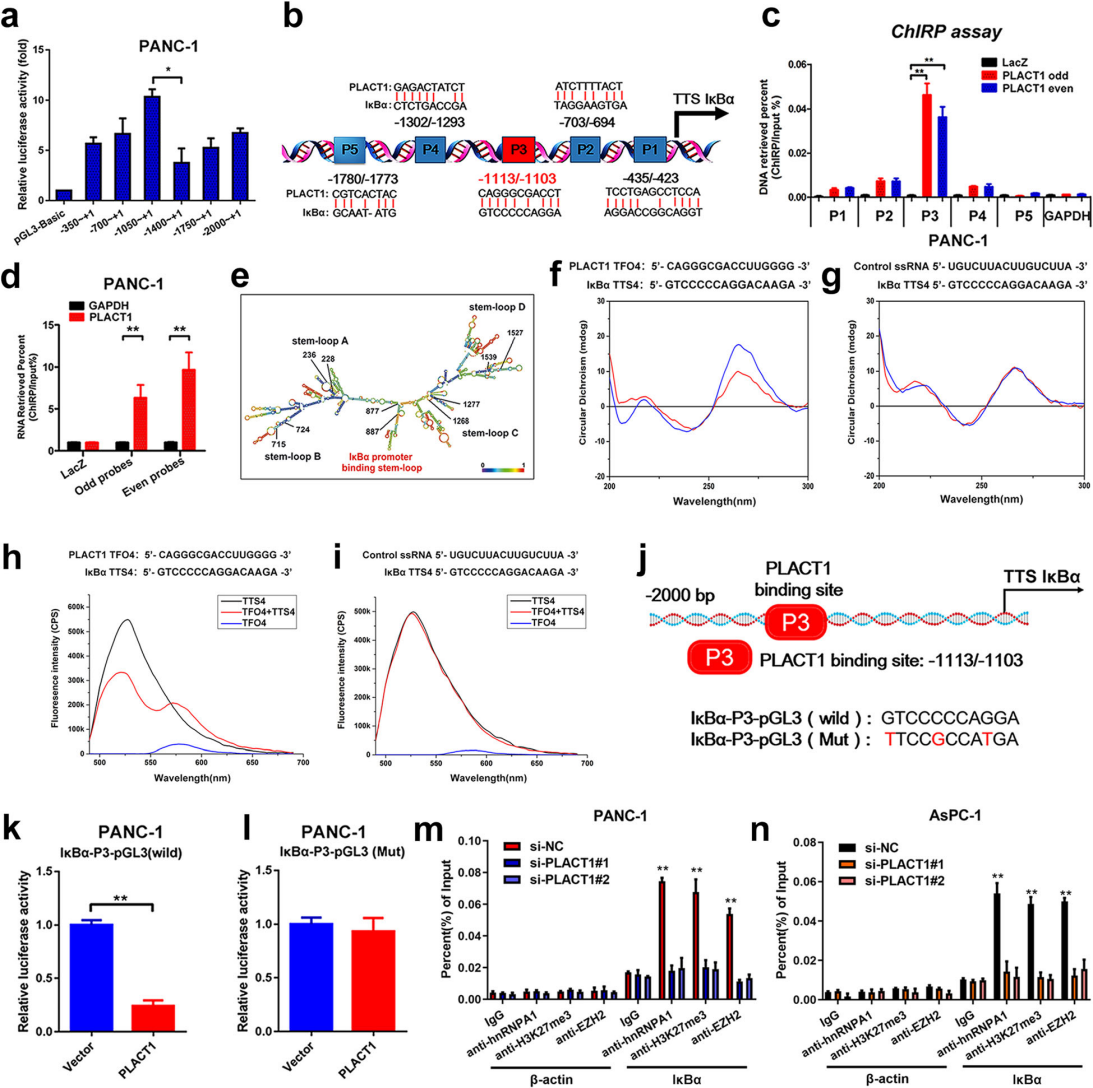
*PLACT1* forms triplexes with the promoter of IκBα and downregulates IκBα expression. **a** Luciferase reporter assays and sequential deletions detect transcriptional activity of the IκBα promoter. **b** Schematic images of the potential *PLACT1* binding sites in the IκBα promoter. **c-d** ChIRP analysis of *PLACT1*-associated chromatin in PANC-1. Retrieved chromatin and RNA were assessed by qRT-PCR. **e***PLACT1* is predicted to have 5 stable stem-loop structures (http://rna.tbi.univie.ac.at/). The red text indicates the IκBα promoter binding stem-loop structures in *PLACT1*. **f-g** CD spectroscopy of the mixture (blue) and the sum (red) of TFO in *PLACT1* and TTS in the IκBα promoter sequences are shown (**f**). Control ssRNA/ IκBα is used as negative control (**g**). **h-i** FRET of TFO in *PLACT1* (black), TTS in the IκBα promoter sequences (blue), and their mixture (red) are shown (**h**). Control ssRNA/ IκBα is used as negative control (**i**). **j** IκBα promoter with mutated *PLACT1* binding sites and wild-type IκBα promoter were cloned into pGL3-luc reporter vector. **k-l**, Dual-Luciferase reporter assays were performed to analyze IκBα promoter with wild-type (**k**) and mutated *PLACT1* binding site IκBα promoter (**l**). **m-n** ChIP-qPCR analysis of hnRNPA1, EZH2 occupancy and H3K27me3 status in the IκBα promoter after knockdown of *PLACT1* in PANC-1 (**m**) and AsPC-1 (**n**) cells. Statistical significance was calculated by using two-tailed *t*-tests and ANOVA followed by Dunnett’s tests for multiple comparison. The error bars represent triplicate standard deviations. *p < 0.05 and **p < 0.01

To explore the molecular mechanisms by which *PLACT1* impaired IκBα expression, we constructed a series of plasmids with IκBα promoter truncations from -2000 nt to + 1 nt, which were subsequently subjected to luciferase assays. The luciferase activity critically decreased when plasmids containing − 1400 to -1050 bp fragments were transfected (Fig. 7a, b and Additional file 9: Figure S7a).

Furthermore, whether *PLACT1* directly interacted with the promoter region of IκBα was validated by ChIRP assays. The results indicated an enrichment of IκBα promoter fragments (− 1113 to -1103 bp, Fig. 7c, d and Additional file 9: Figure S7b, c) in *PLACT1* (+ 877 to + 887 nt, Fig. 7e), suggesting that a triplex structure was formed between *PLACT1* and IκBα promoter. To further confirm the binding sites between *PLACT1* and IκBα promoter, CD spectroscopy and FRET analysis were performed using synthesized predicted triplex-forming oligonucleotides (TFOs) in *PLACT1* and triplex target sites (TTSs) in IκBα promoter. Compared with the NC group, CD spectroscopy showed distinct peaks at approximately 210 nm and 270-280 nm in the *PLACT1* (TFO4, + 877 to + 887 nt)/ IκBα (TTS4, − 1113 to -1103 bp) group (Fig. 7f, g), which were like those of the positive control group (Additional file 9: Figure S7d). Consistently, FRET analysis showed that the fluorescence intensity significantly increased at 570–580 nm and decreased at approximately 520 nm in the *PLACT1* (TFO4, + 877 to + 887 nt)/ IκBα (TTS4, − 1113 to -1103 bp) group (Fig. 7h, i and Additional file 9: Figure S7e). Together, our data suggest that *PLACT1* downregulates IκBα transcription through DNA-RNA triplex formation with the IκBα promoter sequences.

### *PLACT1* promotes H3K27 trimethylation at the IκBα promoter by interacting with hnRNPA1

To clarify whether *PLACT1* impaired the transcriptional activity of IκBα, we generated an IκBα-promoter mutation-containing pGL3 vector (Fig. 7j). The luciferase assays demonstrated that the mutant IκBα-pGL3 vector significantly increased luciferase activity of IκBα promoter compared the wild-type IκBα-pGL3 vector after co-transfection with *PLACT1* (Fig. 7k, l and Additional file 9: Figure S7f, g). Previous studies have reported that hnRNPA1 interacts with repressive complexes of the Polycomb-group (PcG) [26]. Considering EZH2, the catalytic subunit of Polycomb Repressive Complex 2 (PRC2), was responsible for the histone H3 lysine 27 trimethylation (H3K27me3) and induced transcriptional interference, we further analyzed whether hnRNPA1 could mediate the H3K27me3 on the promoter of IκBα. In this study, we showed that IκBα expression was upregulated in hnRNPA1-silencing PDAC cells (Additional file 9: Figure S7h). Moreover, ChIP analysis indicated that a high level of H3K27me3 and EZH2 was specifically localized at the *PLACT1* binding region in the IκBα promoter by interacting with hnRNPA1 (Fig. 7m, n). Furthermore, hnRNPA1 silencing restored *PLACT1*-induced impairment of IκBα expression (Additional file 9: Figure S7i). Taken together, these data indicate that *PLACT1* downregulates IκBα expression through PRC2-induced H3K27me3 in an hnRNPA1-dependent manner.

### *PLACT1* sustains the NF-κB signaling pathway activation by forming a positive feedback loop with E2F1

**Fig. 8:**
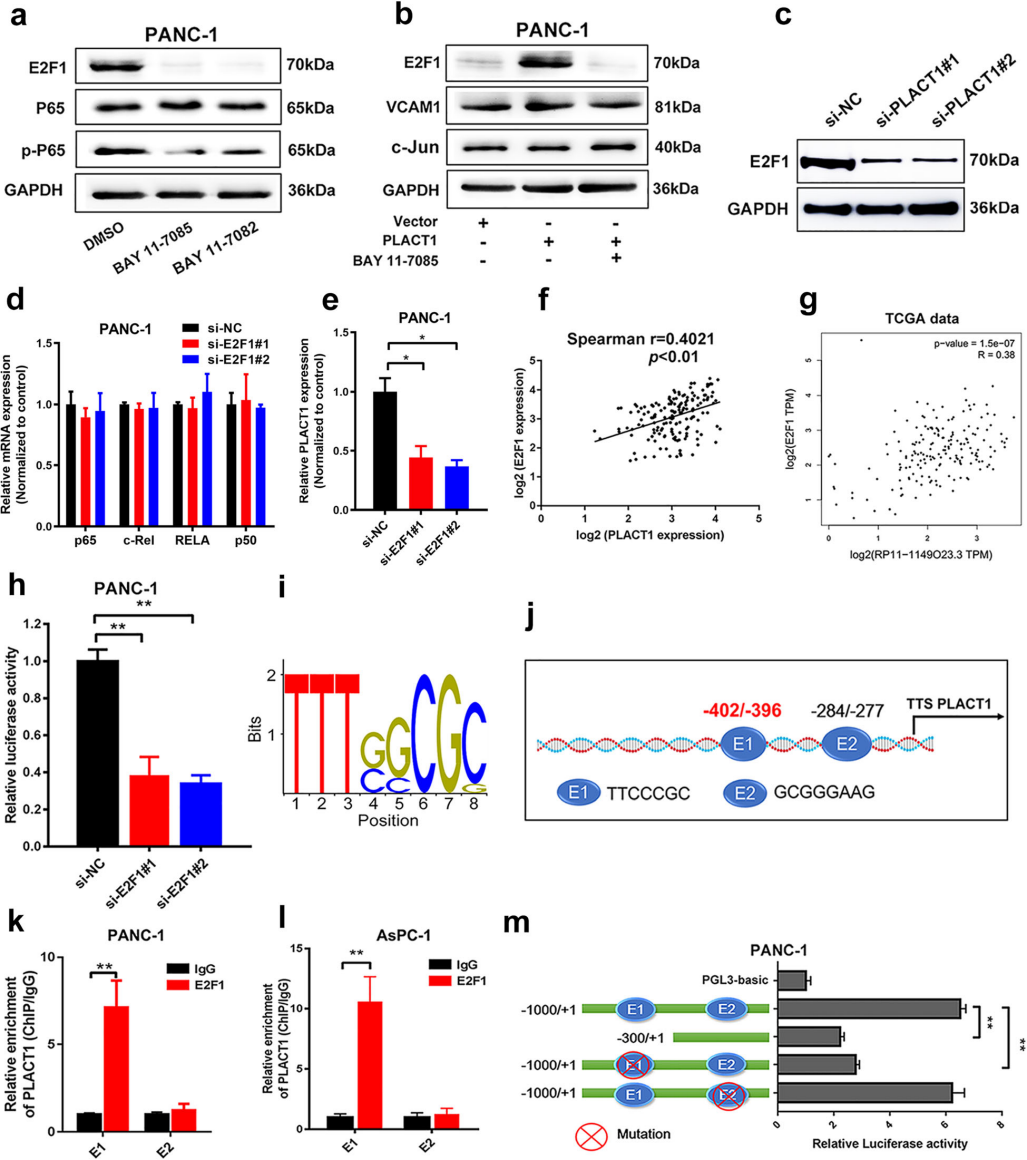
*PLACT1* sustains NF-κB pathway activation by forming a positive feedback loop with E2F1. **a** Western blotting analysis revealed that BAY 11–7085 and BAY 11–7082 decreased E2F1 and p-p65 expression. BAY 11–7085 and BAY 11–7082 are NF-κB inhibitors. **b** Western blotting analysis showed that BAY 11–7085 was used in *PLACT1*-overexpressing cells, and the levels of E2F1, VCAM1, and C-Jun were measured after 72 h. **c** The expression of E2F1 after *PLACT1* knockdown in PDAC cells was assessed by western blotting analysis. **d** qRT-PCR assays showed that E2F1 depletion failed to influence the expression of P65, c-Rel, RELB, and P50 in PANC-1 cells. **e** E2F1 depletion reduced expression of *PLACT1* in PANC-1 cells as detected by qRT-PCR. **f-g***PLACT1* was positively correlated with E2F1 in PDAC tissues evaluated by our data (f, *n* = 166) and TCGA data (g, *n* = 179). **h** Luciferase reporter assays showed that E2F1 knockdown reduced the transcriptional activity of *PLACT1* promoter in PANC-1 cells. **i** Enriched motifs of E2F1 binding sites predicted by JASPAR (http://jaspar.binf.ku.dk/). **j** Schematic model of predicted E2F1 binding sequences in the *PLACT1* promoter region. **k-l** ChIP-qPCR assays were evaluated in PANC-1 (**k**) and AsPC-1 (**l**) cells. **m** Luciferase reporter assays showing that depletion of E1 downregulated the transcriptional activity of *PLACT1* promoter in PANC-1 cells. Statistical significance was evaluated by using two-tailed *t*-tests and ANOVA, followed by Dunnett’s tests for multiple comparison. The error bars represent standard deviations of three independent experiments. *p < 0.05 and **p < 0.01

As expected, *PLACT1* participated in the activation of the NF-κB signaling pathway in an IκBα-dependent manner. However, the downstream genes of IκBα that are associated with PDAC progression remained a mystery. E2F1 is well known to be a critical downstream regulator of the NF-κB signaling pathway [27, 28]. Consistently, western blotting assays showed that E2F1 expression was significantly downregulated after treatment with inhibitors of the NF-κB signaling pathway (Fig. 8a). We further evaluated the alteration of E2F1 expression in PDAC cells that ectopically expressed *PLACT1*, which was co-transfected with BAY 11–7085 (Fig. 8). The results demonstrated that inhibition of the NF-κB signaling pathway significantly impaired E2F1 expression compared with the cells ectopically expressing *PLACT1*. Consistently, Western blot assays revealed that *PLACT1* knockdown significantly decreased the protein levels of E2F1 (Fig. 8c). Moreover, we downregulated E2F1 expression in PDAC cells through transfection with siRNA targeted to E2F1 and found that the expression of P65, RELB, c-Rel, and P50 was not affected by E2F1-silencing (Fig. 8d and Additional file 10: Figure S8a, b). These results suggest that E2F1 is an important downstream gene of the NF-κB signaling pathway in PDAC cells.

Formation of the positive feedback loop was essential in cancer progression [29, 30]. Therefore, we further explored whether E2F1 affected *PLACT1* expression at the transcriptional level. The results indicated that knockdown of E2F1 attenuated the expression of *PLACT1*, while overexpressing E2F1 increased the expression of *PLACT1* in PDAC cells. (Fig. 8e and Additional file 10: Figure S8c-e). We also found that expression of E2F1 was positively associated with *PLACT1* expression in a 166-case cohort of PDAC patients (Fig. 8f). A similar result was obtained from the TCGA database (Fig. 8g). As indicated in Fig. 8h and Additional file 10: Figure S8f, luciferase reporter assays showed a decreasing luciferase activity of *PLACT1* promoter in E2F1-silencing cells compared with the NC group. Moreover, bioinformatics analysis of *PLACT1* promoter predicted two potential binding sequences of E2F1, namely E1 and E2 (Fig. 8i, j).

To further verify the direct interaction between E2F1 and the predicted binding site in the *PLACT1* promoter, ChIP analysis was performed to show that E2F1-E1 could directly bind to the *PLACT1* promoter (− 402 bp to -396 bp) (Fig. 8k, l). The results of luciferase activity assays supported that E2F1-induced luciferase expression was obviously suppressed by E1 mutation, while E2 mutation had no effect (Fig. 8m and Additional file 10: Figure S8g), suggesting that transcription factor E2F1 binds to the *PLACT1* promoter in PDAC cells. These data further support the notion that lncRNA-*PLACT1* sustains NF-κB signaling pathway activation by forming a positive feedback loop with E2F1, causing a transition to aggressive phenotypes and poor outcome in PDAC.

### Blockage of NF-κB signaling pathway reverses *PLACT1*-induced PDAC progression in vivo

**Fig. 9:**
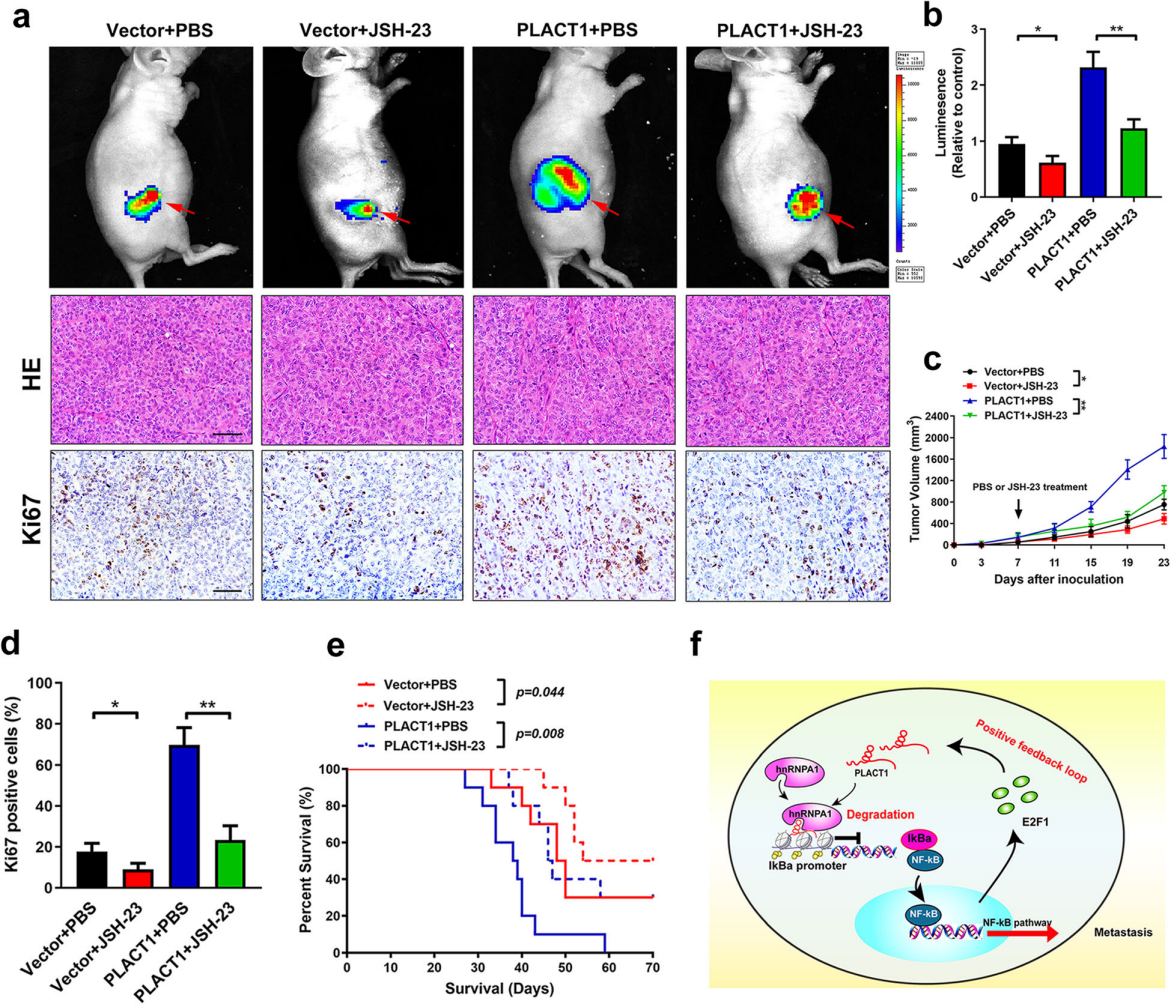
Blocking NF-κB signaling pathway reverses *PLACT1*-induced PDAC progression in vivo*.***a** Representative images of subcutaneous xenografts and IHC for Ki-67. PBS or JSH-23 treatments began 1 week after inoculation (*n* = 10). **b** Histogram analysis of luminescence for subcutaneous xenografts in indicated group (*n* = 10). **c** Tumor volumes were measured in the indicated groups (*n* = 10). **d** Histogram analysis of Ki-67 positive cells in indicated group (*n* = 10). **e** Survival analysis for tumor bearing mice in indicated group (*n* = 10). **f** Model of the role of *PLACT1* forming a positive feedback loop with IκBα/E2F1 in PDAC. Statistical significance was evaluated by using ANOVA, followed by Dunnett’s tests for multiple comparison. The error bars represent standard deviations of three independent experiments. *p < 0.05 and **p < 0.01

Given that *PLACT1*-mediated sustained activation of NF-κB signaling pathway was essential to PDAC development, we further examined whether blocking NF-κB signaling pathway could inhibit *PLACT1*-induced PDAC progression. Overexpression of *PLACT1* promoted the tumor growth in subcutaneous tumor models (*n* = 10 per group) and treatment with JSH-23 significantly reduced *PLACT1*-induced tumorigenicity (Fig. 9a-c). Moreover, compared with PBS treatment, administration of JSH-23 dramatically decreased the Ki-67 level in *PLACT1*-overexpressing PDAC tissues (Fig. 9a, d). In addition, we found that JSH-23 treatment prolonged the survival time of *PLACT1*-transduced tumor bearing mice (Fig. 9e). Taken together, these results suggest that NF-κB signaling pathway inhibition could abrogate *PLACT1*-induced PDAC progression (Fig. 9f).

## Discussion

The proportion of PDAC in pancreatic malignancies has increased to 90%, and the 5-year survival rate is less than 9% [3, 4]. Hence, it is an urgent need to clarify the molecular mechanisms underlying progression of PDAC and provide evidence for novel therapeutic targets. In the present study, we identified a lncRNA, *PLACT1*, which was overexpressed in a cohort of 166 PDAC cases. *PLACT1* knockdown significantly inhibited the proliferation, migration, and invasion of PDAC both in vitro *and* in vivo. Mechanistically, *PLACT1* decreased IκBα transcription by promoting hnRNPA1-induced H3K27me3 on the IκBα promoter. Importantly, we demonstrated that *PLACT1* modulated the progression of PDAC by sustained activation of the NF-κB signaling pathway via an IκBα/E2F1 positive feedback loop. Blocking the activation of NF-κB signaling pathway with NF-κB signaling pathway inhibitor remarkably suppressed the *PLACT1*-induced progression of PDAC in vivo. Our findings provide novel insight into clarifying the regulation mechanism of *PLACT1* in PDAC and inhibition of NF-κB signaling pathway may represent a potential therapeutic strategy for *PLACT1*-overexpressing PDAC patients.

HnRNPA1 is an essential RNA- and DNA-binding protein that directly regulates the alternative splicing isoforms or mediating transcription of target genes [31, 32]. Redon et al. found that hnRNPA1 alleviates the TERRA-mediated inhibition of telomerase by binding to TERRA [33]. Wang et al. reported that hnRNPA1 interacts with lncRNA-lncSHGL to enhance the translation efficiency of CALM, leading to repression of the mTOR/SREBP-1C pathway in hepatocytes [34]. In the present study, we used RNA pull-down and RIP assays to demonstrate that *PLACT1* interacted with hnRNPA1. Moreover, *PLACT1* recruited hnRNPA1 to form a DNA-RNA triplex with IκBα promoter, and suppressed IκBα expression by mediating H3K27me3, which led to activation of the NF-κB signaling pathway. Our data suggest that recruitment by lncRNAs might decide target gene regulation in PDAC cells. *PLACT1*, identified as a functional binding partner of hnRNPA1, regulates the NF-κB signaling pathway through a novel mechanism, which might be crucial to development of PDAC.

Recent studies demonstrated that NF-κB signaling was negatively modulated and terminated by several regulatory factors, including CYLD [35] and IκBs [36]. Indeed, IκBα is impressive for blockage of NF-κB signaling through sequestering NF-κB into the cytoplasm. The degradation of IκBα results in activation of the NF-κB signaling pathway [37]. lncRNAs could guide transcriptional factors or histone protein modification enzymes to specific genomic loci, which lead to inactivation or activation of genes [38]. For example, Grote et al. considered the lncRNA Fendrr, which is implicated in regulation of murine mesoderm differentiation and has recently been reported to form triplexes on the promoter sequence of two target genes: Foxf1 and Pitx2 [39, 40]. Chen et al. reported that lncRNA LNMAT1 upregulated CCL2 expression by associating with the CCL2 promoter via formation of a DNA-RNA triplex in bladder cancer [15]. These studies suggest that formation of DNA-RNA triplexes may be common in lncRNA-mediated transcriptional activation. In the present study, we found that *PLACT1* directly formed a DNA-RNA triplex with the promoter sequences of IκBα. The overexpression of *PLACT1* dramatically increased H3K27 methylation of the promoters of IκBα and significantly inhibited IκBα expression. In addition, *PLACT1* sustained the activation of the NF-κB signaling pathway by attenuating the hnRNPA1/ IκBα axis, suggesting the essential function of *PLACT1* in PDAC initiation.

Sustained activation of NF-κB signaling pathway is a common event in various cancers, including PDAC, and considered to be essential to cancer development. Chen et al. showed that PLCE1 constitutively activated the NF-κB signaling pathway to drive esophageal carcinoma angiogenesis and proliferation [41]. Jeong et al. reported that miR-196b/Meis2/PPP3CC axis sustained the activation of NF-κB signaling pathway to induce prostate cancer castration resistance [42]. However, the mechanism underlying *PLACT1*-induced sustained activation of NF-κB signaling pathway remains to be determined. We previously reported that E2F1 enhanced the transcriptional activity of lncRNA and formed a positive loop to constitutively activate PI3K/Akt pathway [43]. In the present study, we found that E2F1 directly binds to the promoter of *PLACT1* to activate its expression, providing a positive feedback loop that sustains NF-κB activity to promote proliferation, migration, and invasion in PDAC cells. Collectively, *PLACT1* forms a positive feedback loop with IκBα/E2F1, which plays an important role in sustained activation of the NF-κB signaling pathway in PDAC.

NF-κB signaling pathway inhibitors have shown promising effects in suppressing progression of multiple cancers. Lu et al. found that blocked NF-κB signaling pathway using Pristimerin induced apoptosis of chronic myelogenous leukemia cells [44]. Marquardt et al. showed that Curcumin restrained stemness of liver cancer via inhibition of NF-κB signaling pathway [45]. However, lack of specific indicators for cancer treatment is one of the most critical issues that limits NF-κB signaling pathway inhibitors therapy. Herein, we demonstrated that *PLACT1* overexpression facilitated the proliferation and metastasis of PDAC cells and NF-κB signaling pathway inhibitor significantly impaired *PLACT1*-induced PDAC development. Importantly, treatment with NF-κB signaling pathway inhibitor effectively suppressed the tumorigenesis of *PLACT1*-overexpressing PDAC in vivo. Our findings support that *PLACT1* might be a potential indicated marker for clinical intervention with NF-κB signaling inhibitor in PDAC.

## Conclusions

In summary, we provide solid evidence supporting the hypothesis that overexpression of *PLACT1* promotes PDAC progression through sustained activation of the NF-κB signaling pathway by an IκBα/ E2F1 positive feedback loop. Understanding the important role of *PLACT1* in PDAC and activation of the NF-κB signaling pathway will increase our knowledge of the biological basis of PDAC progression and might allow the development of novel therapeutic drugs for patients with PDAC.

## Supplementary information

**Additional file 1:** Supplementary material and methods.

**Additional file 2: **Figure S1. The identification of *PLACT1* in PDAC.

**Additional file 3:** Table S1. Univariate and multivariate analysis of disease-free survival in PDAC patients.

**Additional file 4: **Figure S2. *PLACT1* is overexpressed in multiple types of human cancers.

**Additional file 5: **Figure S3. *PLACT1* enhances proliferation, migration, and invasion of PDAC cells.

**Additional file 6: **Figure S4. *PLACT1* promotes proliferation, migration, and invasion of PDAC cells independent of KRAS/p53.

**Additional file 7: **Figure S5. hnRNPA1 is required for *PLACT1*-induced PDAC progression.

**Additional file 8: **Figure S6. *PLACT1* induces activation of the NF-κB signaling pathway in an IκBα-dependent manner.

**Additional file 9: **Figure S7. *PLACT1* forms triplexes with promoter sequences of IκBα and regulates its expression.

**Additional file 10: **Figure S8. *PLACT1* forms a positive feedback loop with E2F1.

**Additional file 11:** Clinical information on the patient cohort.

**Additional file 12:** Table S2. Primer and probes of experiments.

**Additional file 13:** Table S3. Antibodies of experiments.

**Additional file 14:** Figure S9. Full uncut original pictures.

**Additional file 15: **Table S4. The possible TFO and TTS predicted for *PLACT1* and IκBα promoter.

**Additional file 16: **The original expression data of *PLACT1* in TCGA dataset.

## Data Availability

Our lncRNA microarray datas used in this study have been deposited in NCBI’s Gene Expression Omnibus and are accessible through GEO accession number GSE61166 (https://www.ncbi.nlm.nih.gov/geo/query/acc.cgi?acc=GSE61166).

## Abbreviations

^18^FDG: ^18^F-fluorodeoxyglucose
CCK-8: Cell counting kit-8
CD: Circular dichroism
ChIP: Chromatin immunoprecipitation
ChIRP: Chromatin isolation by RNA purification
DFS: disease-free survival
E2F1: E2F transcription factor 1
FISH: Fluorescence in situ hybridization
FRET: Fluorescence resonance energy transfer
H3K27me3: Histone H3 lysine 27 tri-methylation
hnRNPA1: Heterogeneous nuclear ribonucleoprotein A1
IHC: Immunohistochemistry
IKK: IκB kinase
ISH: In situ hybridization
IκB: Inhibitory κB
IκBα: Inhibitory κBα
LN: Lymph node
lncRNA: Long non-coding RNA
MS: Mass spectrometry
NAT: Normal adjacent tissues
NF-κB: Nuclear factor κB
OS: Overall survival
PDAC: Pancreatic ductal adenocarcinoma
PET-CT: Positron emission tomography and computed tomography
*PLACT1*: Pancreatic cancer associated transcript 1
PRC2: Polycomb repressive complex 2
RACE: Rapid amplification of cDNA ends
RIP: RNA Immunoprecipitation
shRNA: Short hairpin RNA
siRNA: Small interfering RNA
TCGA: The cancer genome atlas
TFO: Triplex-forming oligonucleotides
TTS: Triplex target sites
