# Correction: lncRNA-PLACT1 sustains activation of NF-κB pathway through a positive feedback loop with IκBα/E2F1 axis in pancreatic cancer

**DOI:** 10.1186/s12943-022-01585-x

**Published:** 2022-06-10

**Authors:** Xiaofan Ren, Changhao Chen, Yuming Luo, Mingyang Liu, Yuting Li, Shangyou Zheng, Huilin Ye, Zhiqiang Fu, Min Li, Zhihua Li, Rufu Chen

**Affiliations:** 1 Department of Medical Oncology, Sun Yat-sen Memorial Hospital, State Key Laboratory of Oncology in South China, 107th Yanjiangxi Road, Yuexiu District, Guangzhou, Guangdong province 510120 People’s Republic of China; 2 Guangdong Provincial Key Laboratory of Malignant Tumor Epigenetics and Gene Regulation, Sun Yat-sen Memorial Hospital, State Key Laboratory of Oncology in South China, 107th Yanjiangxi Road, Yuexiu District, Guangzhou, 510120 Guangdong province People’s Republic of China; 3 Department of Urology, Sun Yat-sen Memorial Hospital, State Key Laboratory of Oncology in South China, 107th Yanjiangxi Road, Yuexiu District, Guangzhou, Guangdong province 510120 People’s Republic of China; 4 Department of Hepatopancreatobiliary Surgery, Sun Yat-sen Memorial Hospital, 107th Yanjiangxi Road, Yuexiu District, Guangzhou, 510120 Guangdong province People’s Republic of China; 5 Department of Medicine, Department of Surgery, the University of Oklahoma Health Sciences Center, 975 NE 10th Street, BRC 1262A, Oklahoma City, OK 73104 USA; 6 Department of General Surgery, Guangdong Provincial People’s Hospital, Guangdong Academy of Medical Sciences, 106th of 2nd Zhongshan Road, Yuexiu District, Guangzhou, Guangdong Province 510080 People’s Republic of China

**Correction: Mol Cancer 19, 35 (2020)**

**https://doi.org/10.1186/s12943-020-01153-1**

Following publication of the original article [1], errors were identified in the images presented in Figs. 2 and 6; specifically:

Fig. 2K: overlap was found between the Transwell migration assay and invasion assay in si-PLACT1#1 group of AsPC-1 cells; the representative image of the invasion assay in si-PLACT1#1 group of AsPC-1 cells has been replaced with the correct image 
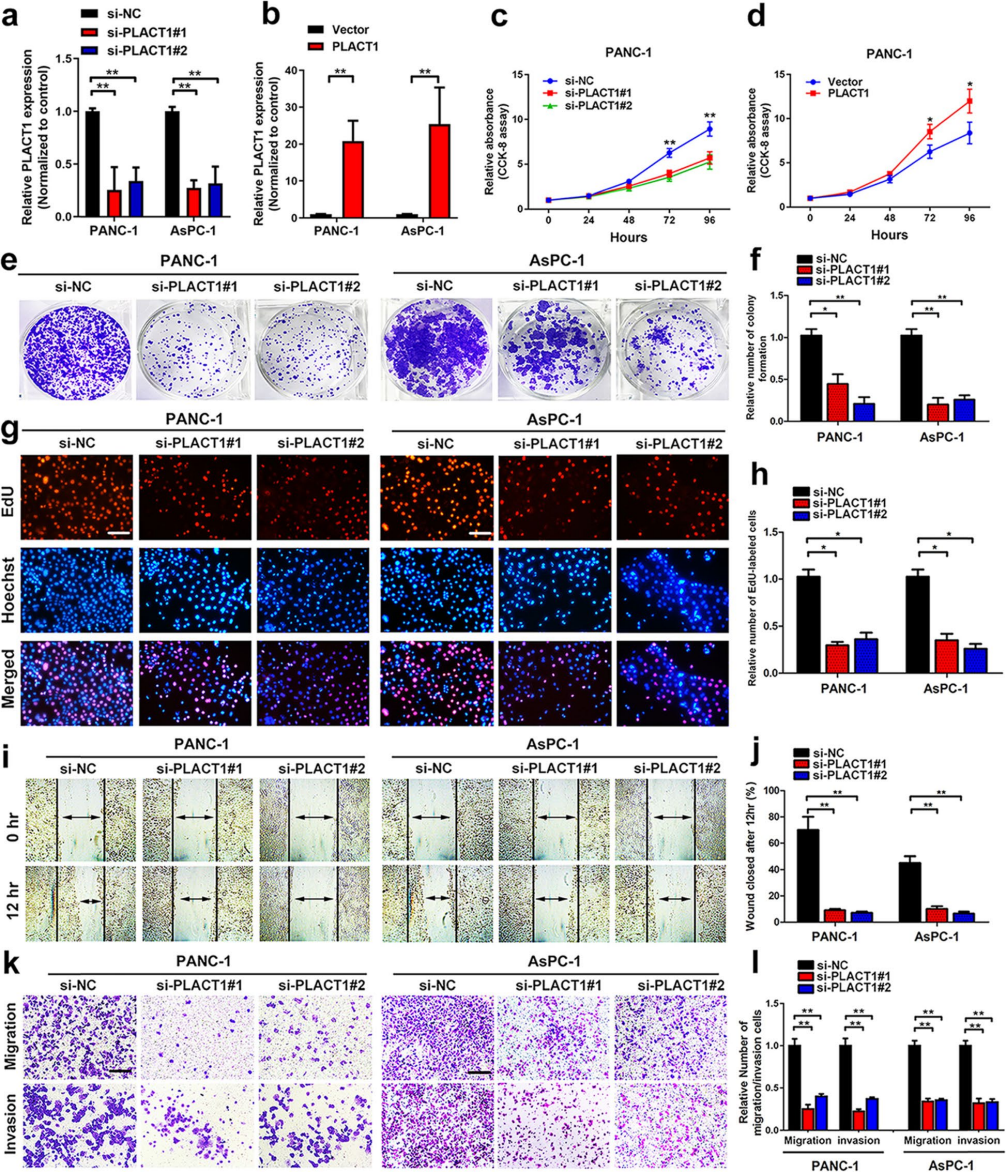


Fig. 6K: overlap was found between the Transwell invasion assay Vector group and PLACT1+JSH-23 group; the correct image is now used (column 1, row 2) 
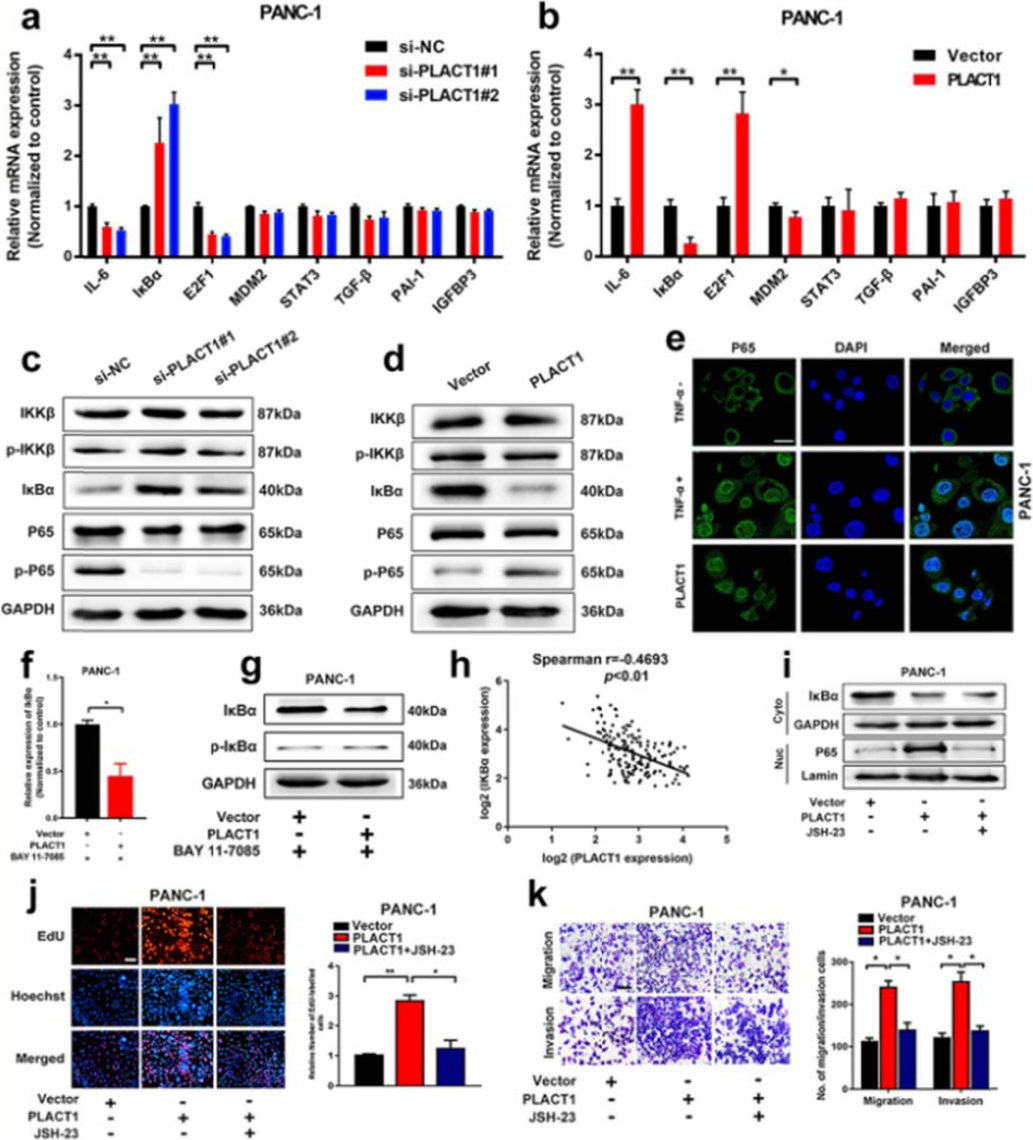


The corrected figures are provided here. The correction does not have any effect on the results or conclusions of the paper. The original article has been corrected.
