# Supplementary material for: lncRNA-PLACT1 sustains activation of NF-κB pathway through a positive feedback loop with IκBα/E2F1 axis in pancreatic cancer
Source: Mol Cancer. 2020 Feb 21;19:35. doi: 10.1186/s12943-020-01153-1 (PMC7033942; doi:10.1186/s12943-020-01153-1)
Supplement: Supplementary file 1 — Additional file 1: Supplementary material and methods. [file 12943_2020_1153_MOESM1_ESM.docx]

**Supplementary methods**

***Patients and clinical samples***

A total of 166 paired primary PDAC specimens were obtained from patients who underwent surgery at Sun Yat-sen Memorial Hospital of Sun Yat-sen University between February 2008 and February 2018. For RNA and protein extraction, tissue specimens were immediately frozen in liquid nitrogen and stored at −80 °C until required. For immunohistochemistry (IHC), tissue specimens were fixed in 10% (v/v) neutral-buffered formalin, dehydrated in 70% ethanol and embedded in paraffin. Studies using human specimens were approved by The Hospital's Protection of Human Subjects Committee. No patients received radiotherapy or chemotherapy before surgery. The surgical specimens and diagnosis of PDAC were confirmed consistently by two independent professional pathologists. The clinical information on the patient cohort are listed in Additional file 11. The detailed clinical pathologic characteristics of patients are summarized in Table 1.

***ISH and IHC analysis***

ISH experiments were performed to evaluate PLACT1 expression. Briefly, after dewaxing and rehydration, the samples were digested with 20μg/ml proteinase K, fixed in 4% paraformaldehyde, and washed with distilled water. Samples were then hybridized with double (5’ and 3’)-digoxin-labeled PLACT1 probe (Exiqon) at 42°C overnight, and incubated overnight at 4°C with anti-digoxin monoclonal antibody conjugated to alkaline phosphatase. The samples were observed after staining with nitro blue tetrazolium/5-bromo-4-chloro-3-indolylphosphate (Roche). As for IHC experiments, paraffin-embedded samples were stained for Ki67 or IκBα. The sections were blocked in normal goat serum for 30 min and incubated with primary antibody at 4°C overnight. The avidin-biotin peroxidase detection systems with the DAB substrate were used to mark the location of antigen. Then, the nuclei were counterstained with hematoxylin. The PLACT1 expression in PDAC tissues was evaluated by using the histochemical score (H-score). The intensity of staining was multiplied by the percentage of positive cells and the H-score (0~300) of each tissue was obtained for statistical analysis. The samples were classed as low (score <50) or high (score ≥50) expression. Staining was professionally reviewed and scored by two experienced independent pathologists.

***Cell lines and cell culture***

Human PDAC cell lines (AsPC-1 and PANC-1) were purchased from American Type Culture Collection (ATCC, Manassas, USA), and authenticated by STR typing. PANC-1was cultured in DMEM (Invitrogen, USA). AsPC-1 was cultured in RPMI 1640 (Invitrogen, USA). All media was supplemented with 10% fetal bovine serum (FBS; Gibco, USA) and 1% penicillin/streptomycin. Cells were cultured in a humidified atmosphere of 5% CO_2_ at 37°C.

***Lentivirus infection and cell transfection***

To establish stable knockdown cell lines, PLACT1 expression was knocked down by transduction of pLKO.1-Puro. The sequences of shRNAs are listed in Additional file 12: Table S2. All siRNAs used in the study were as follows: PLACT1 siRNA (si-PLACT1), P65 siRNA (si-P65), E2F1 siRNA (si-E2F1), and scrambled siRNA (si-NC), which were gained (GenePharma, Shanghai, China). The siRNAs were transfected using Lipofectamine 3000 (Life Technologies, CA, USA) according to the manufacturer′s instructions. The specific siRNA oligos are shown in Additional file 12: Table S2.

***RNA extraction and qRT-PCR assays***

Total RNA was extracted from PDAC cells using TRIzol Reagent (Takara Biotechnology, China). Total RNA was then transcribed to cDNA using PrimerScript RT-PCR kit (Takara Biotechnology, China). qRT-PCR was conducted using a SYBR Green reaction mix (Qiagen, Germany) with a LightCycler 96 System (Roche). The transcription expression of GAPDH or β-actin was utilized as an internal control. All primers are listed in Additional file 12: Table S2.

***Rapid amplification of cDNA ends (RACE)***

3’ RACE assays were performed following the instructions of a SMARTer RACE kit (CLONTECH Laboratories, CA, USA). The 3’ RACE PCR products were separated on a 1% agarose gel and further subjected to bidirectional sequencing. *PLACT1*-specific nested PCR primers sequences used for 3’ RACE analysis was provided in Additional file 12: Table S2.

***CCK-8, Edu assays, and colony formation***

For CCK-8 assays, PDAC cells were transfected with siRNA or overexpression plasmid, followed by seeding in 96-well plates at a density of 4 x 10^3^ cells per well, and cultured overnight. Cells were treated with CCK-8 solution according to the manufacturer’s instructions from the CCK-8 kit (APExBIO, USA). The number of viable cells was measured by OD450 with a microplate reader (Epoch, BioTek, USA) every 24 h for 4 days.

Cell proliferation was measured with EdU assays, following the instructions. After siRNAs or overexpression plasmid transfection, PDAC cells were incubated in 24-well plates for 24 h. 100μl of EdU was added to the cells, and DAPI was applied to stain the nuclei. The images were obtained with an Olympus laser scanning microscope system.

For the colony formation assays, AsPC-1 or PANC-1 cells transfected with siRNA or overexpression plasmid (at the density of 1000 cells per well) were seeded into 6-well plates. The cells were cultured in a humidified atmosphere containing 5% CO2 at 37 °C for 2 weeks. After washing with phosphate buffered saline (PBS), 0.1% crystal violet staining was used to observe colonies. Visible colonies were then manually counted. Wells were measured in triplicate for each treatment group.

***Wound healing and Transwell assays***

Once PDAC cells in 6-well plates reached 85% confluency, using a 10-μl sterile pipette tip we scratched the cell layer. The wound formation was photographed at two different points (0, 12 hours). The size of the wound was measured at least three times.

For Transwell invasion and migration assays, 5 × 10^4^ cells were plated in 24-well plates (Corning Costar Corp) according to the manufacturer’s instructions. After transfection for 48 hours, cells were collected and suspended in serum-free medium. For migration assays, cells were placed in the upper chambers of non-coated membranes. For invasion assays, the membranes of upper chambers were 1:8 diluted and Matrigel-coated (BD Biosciences, USA). All lower chambers were filled with 500 ul of medium containing 25% FBS. After 24 or 48 hours of incubation at 37°C, the number of cells that migrated or invaded was imaged and was counted in three fields under a 200 × objective lens using a microscope.

***Subcutaneous tumorigenicity, orthotopic xenografts and tail vein injection assays***

The nude mice (4-6 weeks old) were purchased and maintained at the Experimental Animal Center of Sun Yat-sen University in SPF barrier facilities. Institutional guidelines of Guangdong Province and by the Use Committee for Animal Care approved the animal care and experimental protocols. PANC-1 cells stably transfected with sh-PLACT1#1 or sh-NC were re-suspended at 1 x 10^8^ cells/ml and vector or PLACT1 transfected PANC-1 cells were re-suspended at 5 x 10^7^ cells/ml. For subcutaneous tumorigenicity, a total of 100μl of suspended cells were subcutaneously injected into the right bilateral hind legs of mice. For NF-κB inhibitor treatment, mice received intratumorally injection with PBS or JSH-23 (3mg/kg) daily during days 7-20 after tumor transplantation. The size of tumor volume was calculated every four days. For orthotopic xenografts, the nude mice were anesthetized with 4% chloral hydrate (0.1 ml/g dose). The left flank of mice was opened and the spleen was exteriorized to access the pancreas. A total of 5 x 10^6^ sh-PLACT1#1- or sh-NC-transfected PANC-1 cells were orthotopically injected into the head of pancreas. Tumor growth and metastasis was monitored by a SIEMENS Inveon micro PET-CT scanner (Munich, Germany) for small animals 4 weeks after inoculation. For tail vein injection assays, a total of 1 x 10^7^ cells were injected into tail veins of BALB/c nude mice. Animals were sacrificed by cervical dislocation at 28 days post injection. For survival analysis, animals were observed until death or sacrificed 70 days after inoculation. The tumors and lungs were collected for further study. The tumors and lungs of the mice were dissected surgically at the end of the experiment.

***Western blotting analysis***

After separation in 10% SDS–PAGE gel, protein extracts were transferred onto a polyvinylidene fluoride (PVDF) membrane and placed into 5% skim milk for blocking at room temperature for 1 h. Subsequently, primary antibodies were added and incubated overnight at 4°C. Finally, the blots were incubated with secondary antibodies. The antibodies are listed in Additional file 13: Table S3. The proteins were visualized with a Tanon-5200 Chemiluminescent Imaging System (Tanon, China). The full uncut original pictures are shown in Additional file 14: Figure S9.

***RNA pull-down assays***

The full-length sense and antisense of *PLACT1* were prepared by *in vitro* transcription using TranscriptAid T7 High Yield Transcription Kit (Thermo Scientific), and were treated with RNase-free DNase I and purified with a GeneJET RNA purification kit (Thermo Scientific). The RNA pull-down assay was performed with a Magnetic RNA-Protein Pull-Down Kit (Thermo Scientific). The biotin-labeled RNA and protein extract were used in each pull-down assay. For RNA pull-down assay with *in vitro* synthesized protein, the full-length of ORF fragment of a specific gene was inserted into pcDNA3.1 and was *in vitro* transcribed and translated with a 1-Step Human Coupled In Vitro Expression Kit following standard protocols.

***Chromatin isolation by RNA purification (ChIRP) assays***

ChIRP was conducted using the Magna ChIRP^TM^ Chromatin Isolation by RNA Purification Kit (Millipore, USA) according to the manufacturer's instructions. Briefly, 3-end Biotin-TEG modified-DNA probes against *PLACT1* were designed using a single-molecule FISH online designer and divided into odd or even groups. 2 × 10^7^ PDAC cells were collected for each ChIRP assay and crosslinked for each hybrid reaction. The cell lysates were sheared into 100-500bp fragments with mean sizes of 150-200bp and hybridized with probes for 4h at 37°C. Finally, DNA and RNA were isolated and purified from the beads for qRT-PCR.

***RIP assays***

The RIP assays were performed using an EZ-Magna RIP kit (Millipore, Massachusetts, USA) following the manufacturer′s instructions. A total of 1 x 10^7^ PANC-1 or AsPC-1 cells were lysed with RIP lysis buffer. Cell extracts were incubated with target antibodies or negative control normal mouse or rabbit IgG containing magnetic beads. Retrieved RNA was subjected to qRT-PCR analysis. Normal IgG was utilized as a negative control and U1 was utilized as a non-specific control.

***Nuclear-plasma fractionation assays***

Adherent cells were collected with trypsin-EDTA and suspended with PBS. The nuclear and cytosolic fractionation of PANC-1 cells were separated using the NE-PER™ Nuclear and Cytoplasmic Extraction Reagents following the manufacturer′s instructions. Briefly, for plasma fractionation, 1 x 10^7^ cells were harvested and washed with PBS. We added ice-cold CER I and CER II and incubated for 10 min on ice. Then, cells were centrifuged for 5 min at 16,000 x g and the supernatant was kept. We suspended the pellet in ice-cold NER and vortexed for 15 seconds every 10 minutes, for a total of 40 minutes. After centrifuging the tube at 16,000 x g for 10 minutes, we collected the supernatant as nuclear fractionation. The plasma portion and nuclear extract fraction were kept at -80°C until use.

***Immunofluorescence analysis***

The cells were fixed with 3.7% formaldehyde and treated with 0.1% Triton X-100. After washing with PBST (1 x PBS with 0.1% Tween-20) twice, 50 μl of blocking solution (1 x PBS, 1% BSA, 0.1% Tween-20) was added. Then, the cells were [sequential](http://www.youdao.com/w/sequential/#keyfrom=E2Ctranslation)ly incubated with primary antibody at 4°C overnight, then conjugated secondary antibody and DAPI at room temperature. Images were captured with the LSM710 confocal microscope (Zeiss, Pleasanton, CA, USA). All antibodies used are listed in Additional file 13: Table S3.

***FISH analysis***

5 x 10^4^ cells were seeded on a glass-bottomed confocal plate and cultured overnight. After fixation with 4% PFA and permeabilization with 0.5% Triton, hybridization was carried out overnight with the probes conjugated with Cy-3 at 37°C in 2×SSC, 10% formamide and 10% dextran. Finally, the nuclei were stained by DAPI and the images were captured under a confocal microscope.

***CD spectroscopy***

The mixture was prepared by mixing PLACT1 ssRNA TFO (2.2μM) and dsDNA TSS oligos of IκBα promoter sequences (2.2μM) in binding buffer (20mM HEPES pH 7.5, 50mM Na-acetate, 10mM MgCl_2_) and equilibrating for approximately 1 h at 30°C. Control ssRNA/ IκBα TSS was used as NC and FENDRR TFO/PTIX2 TTS served as positive control. The measurements were performed on a Chirascan at room temperature in a -mm cuvette using 200μl of solution for TFO oligos, TTS oligos, and the mixture separately.

***FRET assays***

TFO and TSS oligos were respectively labeled with 5-carboxytetramethylrhodamine (TAMRA) and 5-Carboxyfluorescein (FAM) on the 5′ end. A 1:5 mixture of TSS (500nM) and TFO (2500nM) in binding buffer (20mM HEPES pH 7.5, 50mM Na-acetate, 10mM MgCl_2_) was annealed at 55°C for 10 min and incubated at 37°C for 10h . The fluorescence intensities were evaluated using a Molecular Device M5 Plate Reader (excitation wavelengths: 470 nm, emission wavelengths: 480 and 690 nm). The oligos used in CD spectroscopy and this study are shown in Additional file 15: Table S4.

***Dual-luciferase reporter assays***

The indicated regions of IκBα promoter was directly inserted into pGL3 luciferase reporter plasmid. The pcDNA3.1-E2F1 and pcDNA3.1- PLACT1 were co-transfected into PDAC cells. The pGL3 vector was utilized as a negative control. The luciferase activities were detected using the Dual-Luciferase Reporter Assay System (Promega, USA). Renilla luciferase activity was normalized against Firefly luciferase activity for intra-experimental transfection efficiency.

***ChIP assays***

ChIP assays were conducted using the EZ-Magna ChIP A/G kit (Millipore, Massachusetts, USA). Briefly, stable transfected cells were fixed with 1% formaldehyde for 10 min at room temperature and were then lysed with SDS lysis buffer. Ultrasonication was used to shear the DNA into 200~500nt fragments. Antibodies targeting E2F1 and H3k27me3 were incubated for each assay. The immunoprecipitated DNA fragments were detected by qRT-PCR. Primers are listed in Additional file 12: Table S2.

***Bioinformatics analysis***

The promoter of the PLACT1 was searched in the UCSC database. The website is: <http://www.genome.ucsc.edu/>. The E2F1 binding sites in the promoter of PLACT1 were predicted using the JASPAR database (<http://jaspardev.genereg.net/>).

***Statistical analysis***

All statistical analyses were performed using SPSS 13.0 software (IBM, SPSS, Chicago, IL, USA). Two-tailed Student's t-tests were utilized for comparisons between two groups. One-way ANOVA with Bonferroni's test was utilized for multiple comparisons. The overall survival (OS) and disease-free survival (DFS) were calculated using the Kaplan–Meier method with the log-rank test. A multivariate Cox regression analysis was used to evaluate the survival data. All data are calculated as the mean ± SD from at least three independent experiments. A p-value <0.05 was considered statistically significant.
