# Supplementary material for: lncRNA-PLACT1 sustains activation of NF-κB pathway through a positive feedback loop with IκBα/E2F1 axis in pancreatic cancer
Source: Mol Cancer. 2020 Feb 21;19:35. doi: 10.1186/s12943-020-01153-1 (PMC7033942; doi:10.1186/s12943-020-01153-1)
Supplement: Supplementary file 2 — Additional file 2: Figure S1. The identification of PLACT1 in PDAC. [file 12943_2020_1153_MOESM2_ESM.docx]

**Figure S1**


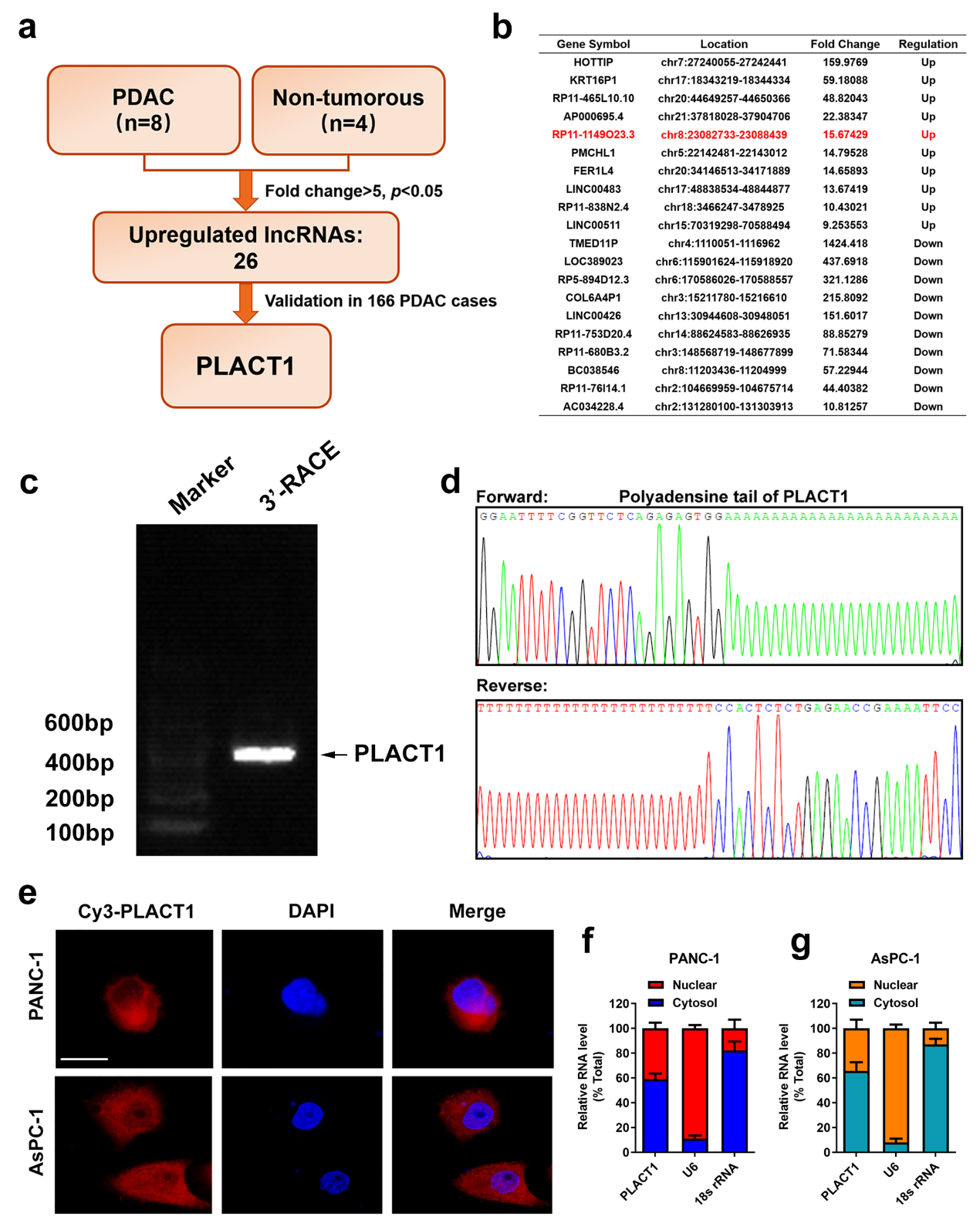


**Figure S1. The identification of *PLACT1* in PDAC. a**, Schematic illustration of the identification of lncRNAs upregulated in PDAC tissues compared with non-tumorous tissues. **b**, The top 10 upregulated and downregulated lncRNAs in PDAC identified from microarray analysis. **c-d**, Representative image of agarose gel electrophoresis (c) and sequencing of 3’-RACE PCR products (d) showed the 3’ terminal of *PLACT1*. **e**, Representative image of FISH analysis showed the subcellular distribution of *PLACT1* in PANC-1 and AsPC-1 cells. Scale bar: 5 μm. **f-g**, Subcellular fractionation assays confirmed the subcellular distribution of *PLACT1* in PANC-1 and AsPC-1 cells. U6 was used as nuclear control and 18S rRNA was used as cytoplasmic control.
