# Supplementary material for: lncRNA-PLACT1 sustains activation of NF-κB pathway through a positive feedback loop with IκBα/E2F1 axis in pancreatic cancer
Source: Mol Cancer. 2020 Feb 21;19:35. doi: 10.1186/s12943-020-01153-1 (PMC7033942; doi:10.1186/s12943-020-01153-1)
Supplement: Supplementary file 3 — Additional file 3: Table S1. Univariate and multivariate analysis of disease-free survival in PDAC patients. [file 12943_2020_1153_MOESM3_ESM.docx]

**Table S1. Univariate and multivariate analysis of disease-free survival (DFS) in PDAC patients (*n*=166)**

| **Variables** | **Univariate analysis** | | | **Multivariate analysis** | | |
| --- | --- | --- | --- | --- | --- | --- |
|  | **HR** | **95%CI** | ***p*-Value** | **HR** | **95%CI** | ***p*-Value** |
| Age | 0.710 | 0.500-1.008 | 0.055 |  |  |  |
| Gender | 0.938 | 0.672-1.311 | 0.709 |  |  |  |
| Differentiation  (moderately or poor vs. well) | 1.298 | 0.824-2.047 | 0.261 |  |  |  |
| T stage  (T3 or T4 vs. T1 or T2) | 1.299 | 0.929-1.817 | 0.127 |  |  |  |
| TNM stage (AJCC)  (stage II or stage III vs. stage I) | 2.214 | 1.469-3.336 | 0.001^**^ | 1.385 | 0.794-2.417 | 0.251 |
| Lymphatic metastasis | 2.147 | 1.507-3.058 | 0.001^**^ | 1.700 | 1.057-2.735 | 0.029^*^ |
| PLACT1 expression | 1.776 | 1.268-2.487 | 0.001^**^ | 1.640 | 1.165-2.310 | 0.005^**^ |

Abbreviations: HR = hazard ratio; 95%CI = 95% confidence interval; T stage = tumor stage; TNM = tumor node metastasis. Cox regression analysis, ^*^ *p* <0.05, ^**^ *p* <0.01.
