# Supplementary material for: lncRNA-PLACT1 sustains activation of NF-κB pathway through a positive feedback loop with IκBα/E2F1 axis in pancreatic cancer
Source: Mol Cancer. 2020 Feb 21;19:35. doi: 10.1186/s12943-020-01153-1 (PMC7033942; doi:10.1186/s12943-020-01153-1)
Supplement: Supplementary file 4 — Additional file 4: Figure S2. PLACT1 is overexpressed in multiple types of human cancers. [file 12943_2020_1153_MOESM4_ESM.docx]

**Figure S2**

**
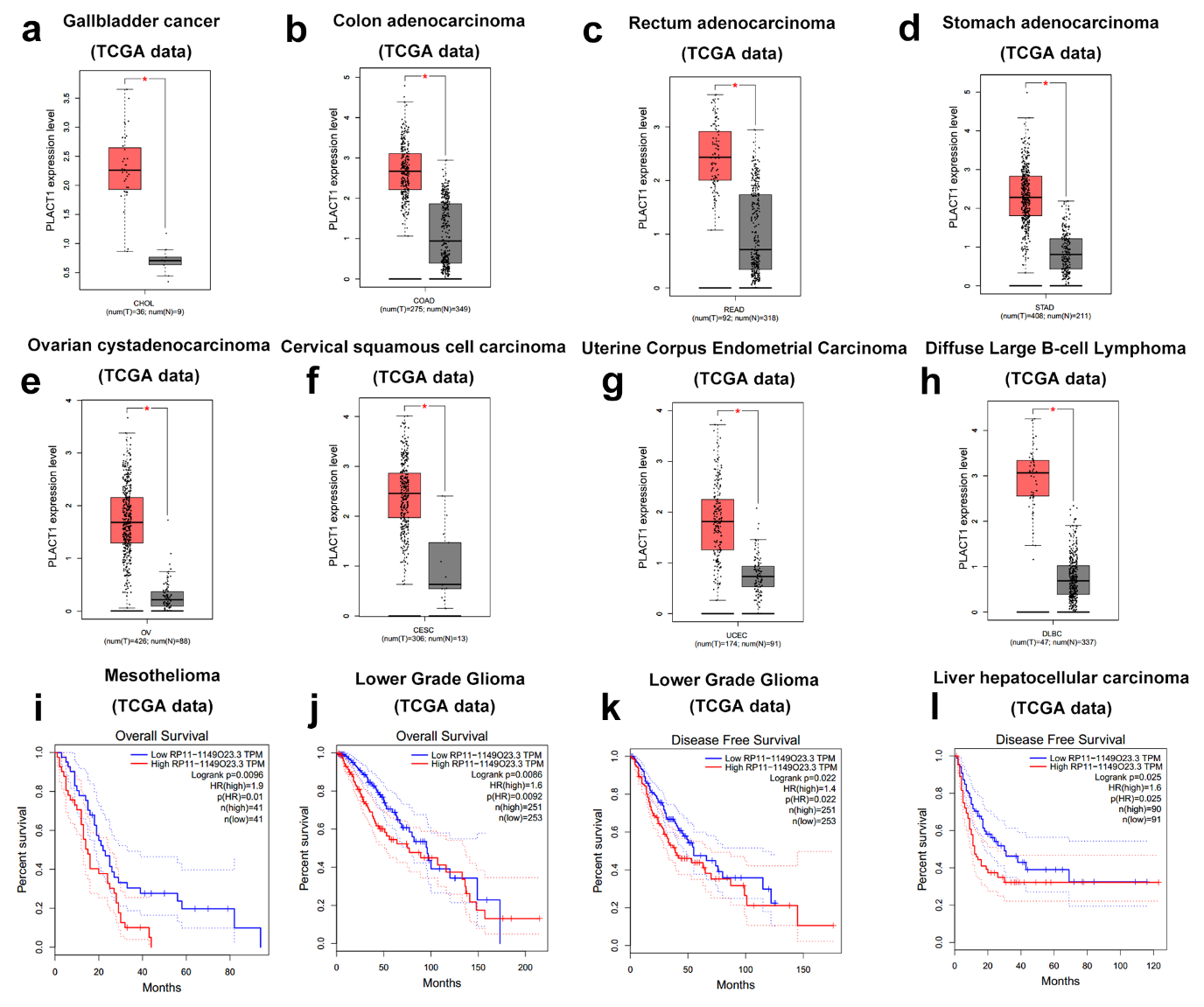
**

**Figure S2. PLACT1 is overexpressed in multiple types of human cancers. a-h**, Data from the TCGA and GTEx datasets for different types of human cancers were analyzed using GEPIA (<http://gepia.cancer-pku.cn/index.html>). The nonparametric Mann-Whitney U test was used. **i-l**, Kaplan-Meier survival analysis of overall survival and disease-free survival for patients with expression profiles of PLACT1-high vs. PLACT1-low in different types of cancers from the TCGA database. The data were obtained from GEPIA. *p*-values were calculated by the log-rank (Mantel-Cox) test. **p* < 0.05 and ***p* < 0.01.
