# Supplementary material for: lncRNA-PLACT1 sustains activation of NF-κB pathway through a positive feedback loop with IκBα/E2F1 axis in pancreatic cancer
Source: Mol Cancer. 2020 Feb 21;19:35. doi: 10.1186/s12943-020-01153-1 (PMC7033942; doi:10.1186/s12943-020-01153-1)
Supplement: Supplementary file 5 — Additional file 5: Figure S3. PLACT1 enhances proliferation, migration, and invasion of PDAC cells. [file 12943_2020_1153_MOESM5_ESM.docx]

**Figure S3**


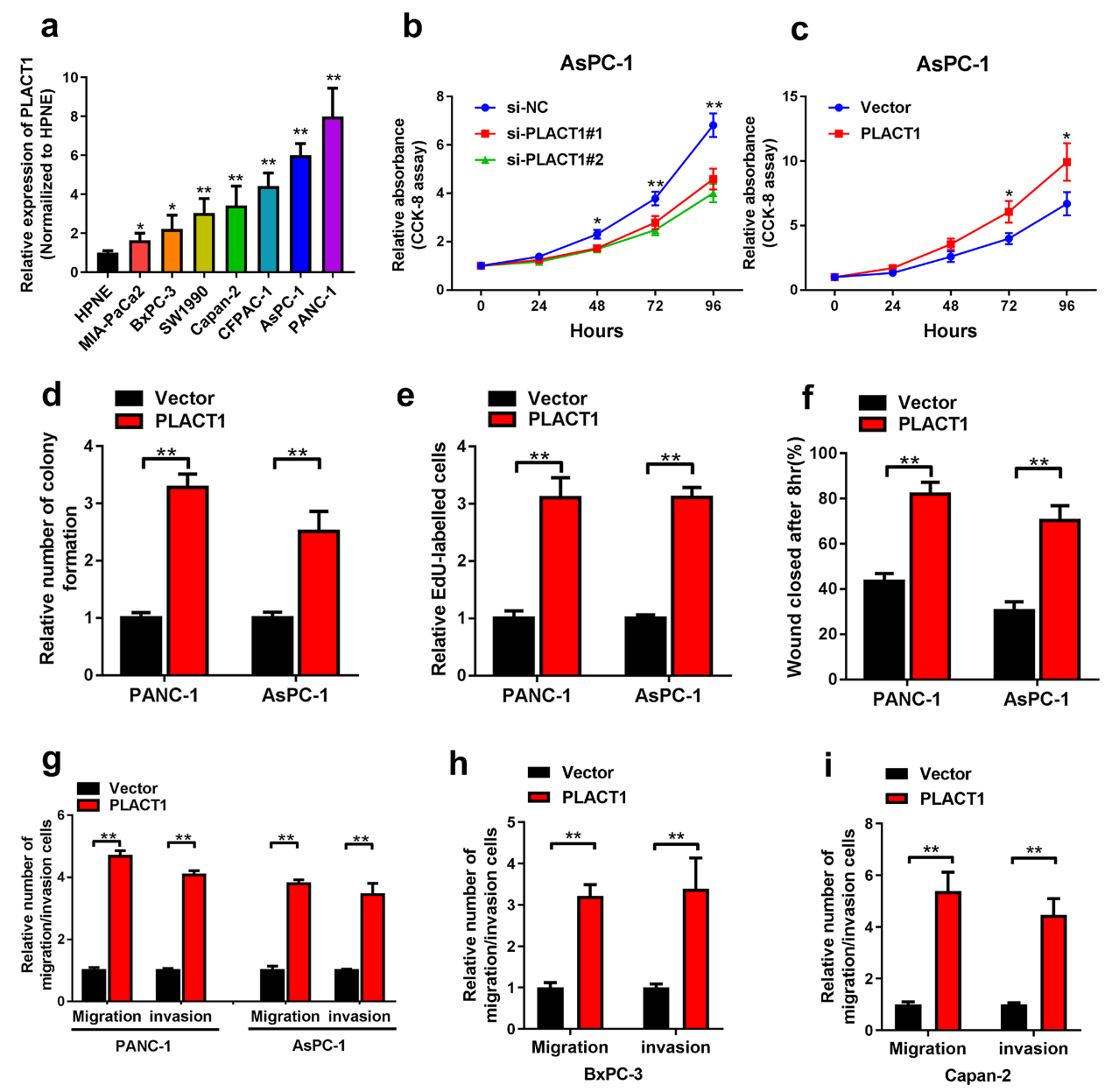


**Figure S3. PLACT1 enhances proliferation, migration, and invasion of PDAC cells. a**, qRT-PCR analyzed the expression of *PLACT1* in PDAC and normal pancreatic ductal epithelial cell lines. **b-c**, The cell viability of si-PLACT1-transfected (b) or PLACT1-cDNA-transfected (c) AsPC-1 cells by CCK-8 assays. **d**, PLACT1 overexpression facilitated colony formation in PANC-1 and AsPC-1 cells. The histogram analysis showed the mean ± *SD* of colonies from three independent experiments. **e**, Histogram analysis of EdU assays after PLACT1 overexpression in PANC-1 and AsPC-1 cells. **f**, Histogram analysis of wound healing assays after PLACT1 overexpression in PANC-1 and AsPC-1 cells. **g**, Histogram analysis of Transwell assays after PLACT1 overexpression in PANC-1 and AsPC-1 cells. **h-i**, Histogram analysis of Transwell assays after PLACT1 overexpression in BxPC-3 (h) and Capan-2 (i) cells. Figures with error bars show standard deviations of three independent experiments. Significance levels were evaluated using two-tailed *t*-tests and ANOVA followed by Dunnett′s tests for multiple comparisons. ***p* < 0.01 and **p* < 0.05.
