# Supplementary material for: lncRNA-PLACT1 sustains activation of NF-κB pathway through a positive feedback loop with IκBα/E2F1 axis in pancreatic cancer
Source: Mol Cancer. 2020 Feb 21;19:35. doi: 10.1186/s12943-020-01153-1 (PMC7033942; doi:10.1186/s12943-020-01153-1)
Supplement: Supplementary file 6 — Additional file 6: Figure S4. PLACT1 promotes proliferation, migration, and invasion of PDAC cells independent of KRAS/p53. [file 12943_2020_1153_MOESM6_ESM.docx]

**Figure S4**


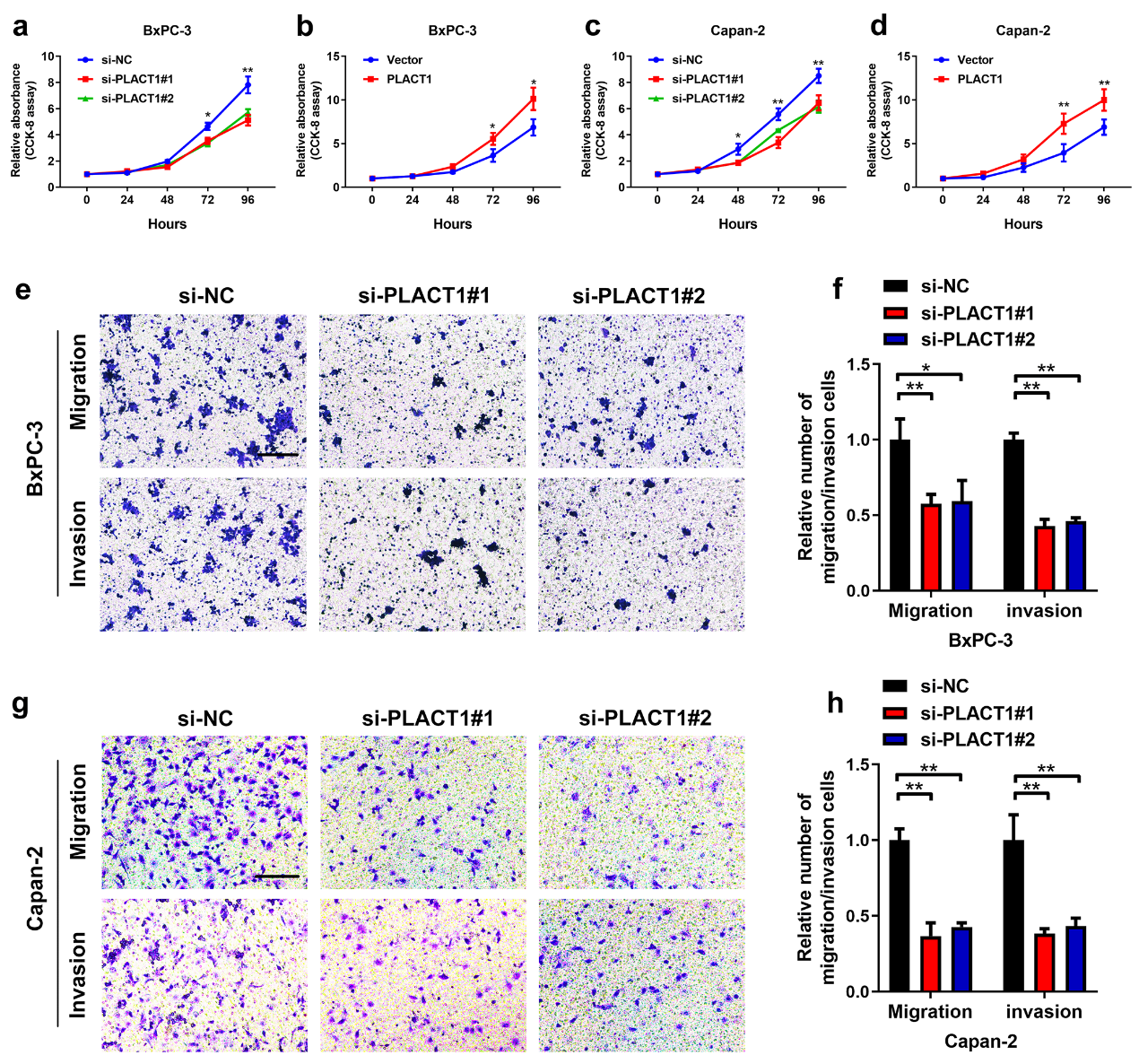


**Figure S4. PLACT1 promotes proliferation, migration, and invasion of PDAC cells independent of** **KRAS/p53. a-d**, CCK-8 assays showed the cell viability of si-PLACT1-transfected (a and c) or PLACT1-cDNA-transfected (b and d) BxPC-3 and Capan-2 cells. **e-h**, Representative images (e and g) and histogram analysis (f and h) of Transwell assays after *PLACT1* knockdown in BxPC-3 and Capan-2 cells. Scale bars: 100 μm. Figures with error bars show standard deviations of three independent experiments. Significance levels were evaluated using two-tailed *t*-tests and ANOVA followed by Dunnett′s tests for multiple comparisons. ***p* < 0.01 and **p* < 0.05.
