# Supplementary material for: lncRNA-PLACT1 sustains activation of NF-κB pathway through a positive feedback loop with IκBα/E2F1 axis in pancreatic cancer
Source: Mol Cancer. 2020 Feb 21;19:35. doi: 10.1186/s12943-020-01153-1 (PMC7033942; doi:10.1186/s12943-020-01153-1)
Supplement: Supplementary file 7 — Additional file 7: Figure S5. hnRNPA1 is required for PLACT1-induced PDAC progression. [file 12943_2020_1153_MOESM7_ESM.docx]

**Figure S5**

**
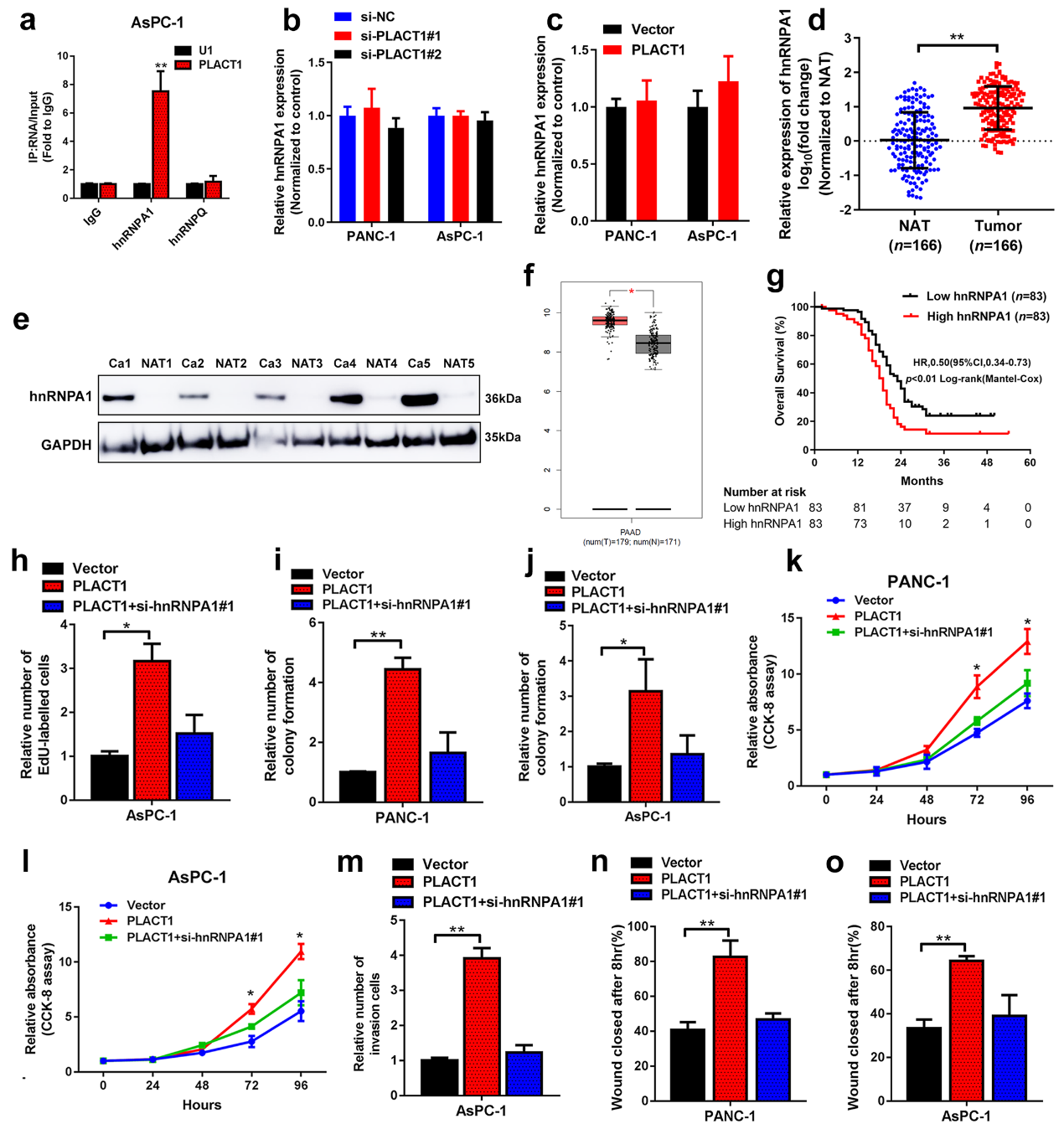
**

**Figure S5. HnRNPA1 is required for PLACT1-induced PDAC progression. a**, RIP assays revealed that PLACT1 bound to hnRNPA1 in AsPC-1 cells. **b-c**, qRT-PCR analysis detected hnRNPA1 expressions in the PLACT1 knockdown (b) or overexpressing (c) cells. **d**, qRT-PCR analysis assessed the expression of hnRNPA1 in human PDAC tissues (n=166) paired with NATs (n=166). The results were determined by nonparametric Mann–Whitney U-test. **e**, Western blotting analysis assessed hnRNPA1 expression in PDAC tissues paired with NATs. **f**, TCGA and Genotype-Tissue Expression (GTEx) data showed the hnRNPA1 expression in PDAC tissues (*n*=179) relative to non-tumorous tissues (*n*=171). The nonparametric Mann-Whitney U test was used. **g**, The Kaplan–Meier curves represented overall survival of PDAC patients with low vs. high expression of hnRNPA1. The cutoff value was the median expression of hnRNPA1. **h-j**, Histogram analysis of EdU (h) and colony formation assays (i and j) revealed that depletion of hnRNPA1 partly reversed the effects of PLACT1-overexpressing PANC-1 and AsPC-1 cells. **k-l**, CCK-8 assays showed that depletion of hnRNPA1 partly reversed the effects of PLACT1-overexpressing PANC-1(k) and AsPC-1(l) cells. **m-o**, Histogram analysis of Transwell (m) and wound healing assays (n and o) revealed that depletion of hnRNPA1 partly reversed the effects of PLACT1-overexpressing PANC-1 and AsPC-1 cells. Significance levels were measured using two-tailed *t*-tests and ANOVA followed by Dunnett′s tests for multiple comparisons. Figures with error bars represent standard deviations of three independent experiments. **p* < 0.05 and ***p* < 0.01.
