# Supplementary material for: lncRNA-PLACT1 sustains activation of NF-κB pathway through a positive feedback loop with IκBα/E2F1 axis in pancreatic cancer
Source: Mol Cancer. 2020 Feb 21;19:35. doi: 10.1186/s12943-020-01153-1 (PMC7033942; doi:10.1186/s12943-020-01153-1)
Supplement: Supplementary file 8 — Additional file 8: Figure S6. PLACT1 induces activation of the NF-κB signaling pathway in an IκBα-dependent manner. [file 12943_2020_1153_MOESM8_ESM.docx]

**Figure S6**


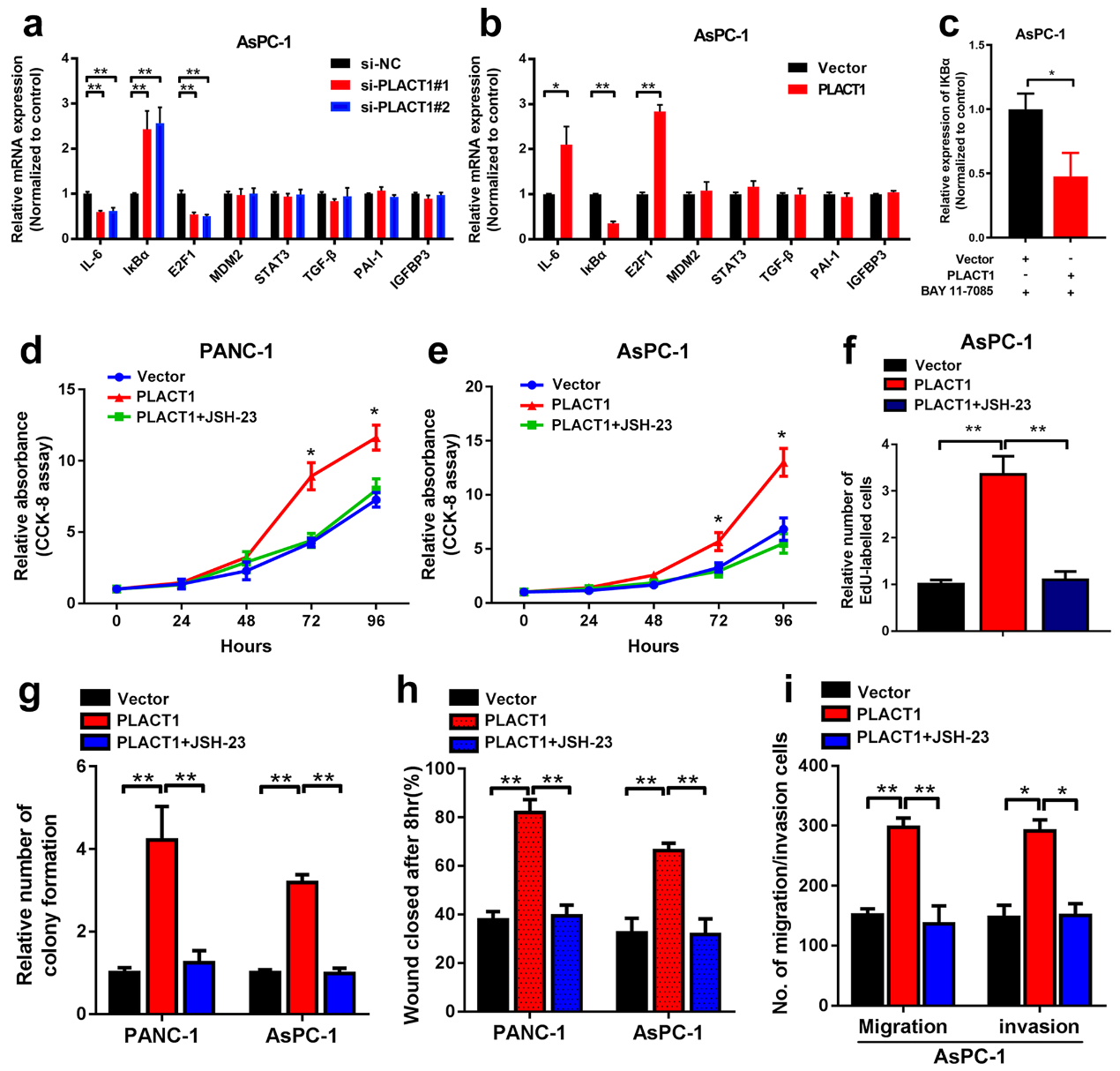


**Figure S6. PLACT1 induces activation of the NF-κB signaling pathway in an IκBα-dependent manner. a-b**, Genes involved in the NF-κB signaling pathway were detected by qRT-PCR in AsPC-1 cells with PLACT1 knockdown (a) or overexpression (b). **c**, qRT-PCR analysis indicated that PLACT1 downregulated the mRNA levels of IκBα in AsPC-1 cells after treatment with BAY 11-7085. **d-e**, CCK-8 assays showed that JSH-23 reversed the effect of PLACT1-overexpressing PANC-1 (d) and AsPC-1 (e) cells. **f**, Histogram analysis of EdU assays showed that JSH-23 reversed the effect of PLACT1-overexpressing AsPC-1 cells. **g-h**, Histogram analysis of colony formation (g) and wound healing assays (h) showed that JSH-23 reversed the effect of PLACT1-overexpressing PANC-1 and AsPC-1 cells. **i**, Histogram analysis of Transwell assays showed that JSH-23 reversed the effect of PLACT1-overexpressing AsPC-1 cells. Statistical significance was assessed by using two-tailed *t*-tests and ANOVA followed by Dunnett′s tests for multiple comparisons. The error bars represent triplicate standard deviations. **p* < 0.05 and ***p*< 0.01.
