# Supplementary material for: lncRNA-PLACT1 sustains activation of NF-κB pathway through a positive feedback loop with IκBα/E2F1 axis in pancreatic cancer
Source: Mol Cancer. 2020 Feb 21;19:35. doi: 10.1186/s12943-020-01153-1 (PMC7033942; doi:10.1186/s12943-020-01153-1)
Supplement: Supplementary file 9 — Additional file 9: Figure S7. PLACT1 forms triplexes with promoter sequences of IκBα and regulates its expression. [file 12943_2020_1153_MOESM9_ESM.docx]

**Figure S7**


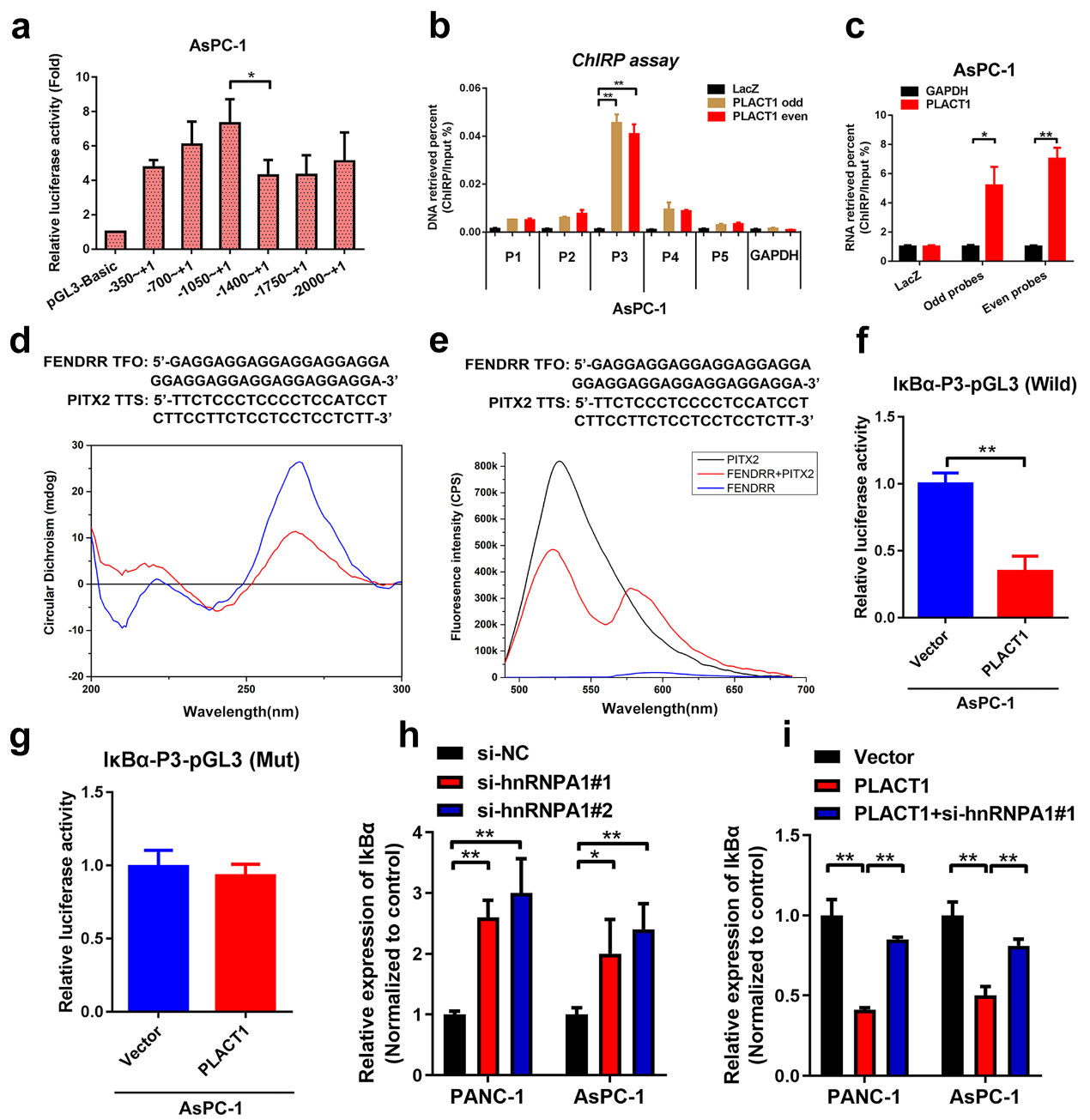


**Figure S7. PLACT1 forms triplexes with promoter sequences of IκBα and regulates its expression. a**, Renilla luciferase activity and sequential deletions were used to assess the transcriptional activity of the IκBα promoter in AsPC-1 cells. **b** and **c**, ChIRP analyzed PLACT1-associated chromatin in AsPC-1 cells. Retrieved chromatin (b) and RNA (c) were assayed by qRT-PCR. **d**, CD spectroscopy of the mixture (blue) and the sum (red) of TFO in FENDRR and TTS in the PITX2 promoter sequences are shown. FENDRR TFO/PITX2 TSS is used as positive control in CD spectroscopy. **e**, FRET of TFO in FENDRR (black), TTS in the PITX2 promoter sequences (blue), and their mixture (red) are shown. FENDRR TFO/PITX2 TSS is used as positive control in FRET assays. **f-g**, Wild-type IκBα promoter (f) and IκBα promoter with mutational PLACT1 binding site (g) were evaluated by dual-luciferase reporter assays in AsPC-1 cells. **h**, qRT-PCR analysis of IκBα expression in the control and hnRNPA1-silenced PDAC cells. **i**, qRT-PCR analysis showed that hnRNPA1 knockdown reversed PLACT1-mediated downregulation of IκBα in PDAC cells. Statistical significance was measured using two-tailed *t*-tests and ANOVA followed by Dunnett′s tests for multiple comparisons. The error bars show standard deviations of three independent experiments. **p* < 0.05 and ***p*< 0.01.
