# Supplementary material for: lncRNA-PLACT1 sustains activation of NF-κB pathway through a positive feedback loop with IκBα/E2F1 axis in pancreatic cancer
Source: Mol Cancer. 2020 Feb 21;19:35. doi: 10.1186/s12943-020-01153-1 (PMC7033942; doi:10.1186/s12943-020-01153-1)
Supplement: Supplementary file 10 — Additional file 10: Figure S8. PLACT1 forms a positive feedback loop with E2F1. [file 12943_2020_1153_MOESM10_ESM.docx]

**Figure S8**

**
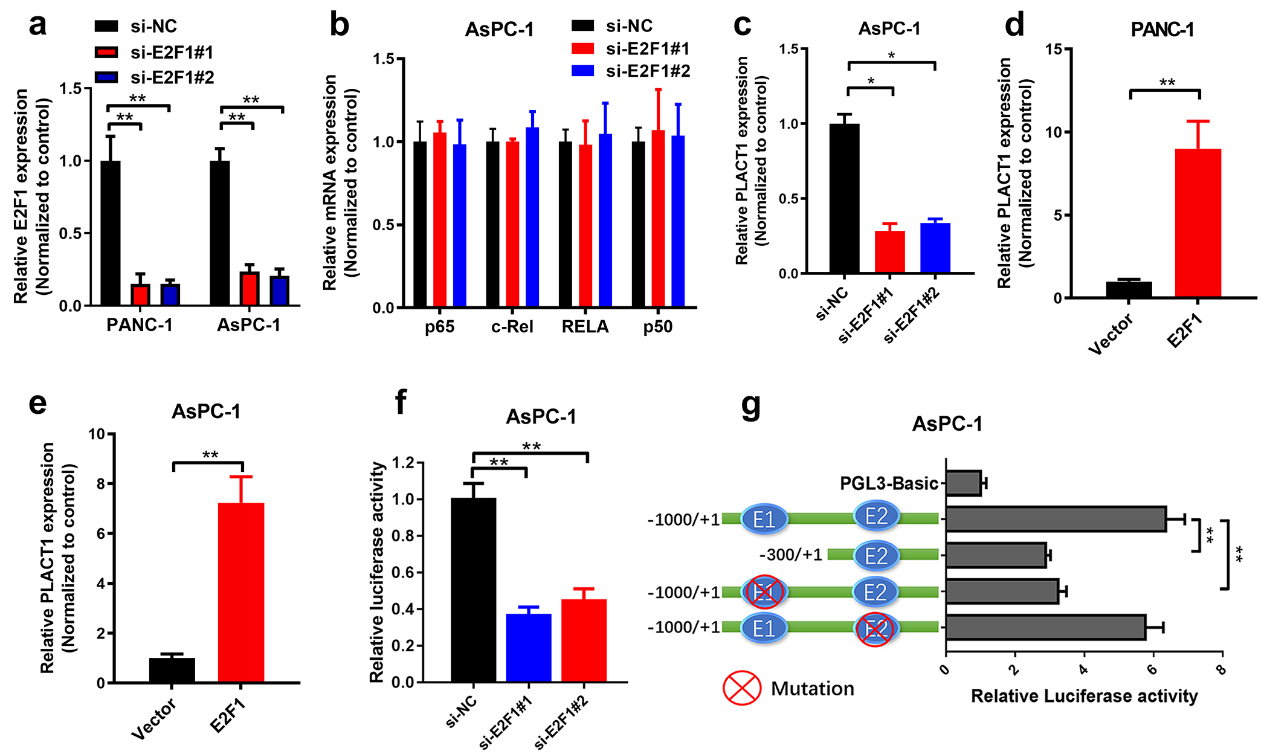
**

**Figure S8. *PLACT1* forms a positive feedback loop with E2F1. a**, Efficiencies of E2F1 knockdown in PANC-1 and AsPC-1 cells were verified by qRT-PCR assays. **b**, qRT-PCR assays showed that E2F1 depletion failed to influence the expression of P65, c-Rel, RELB, and P50 in AsPC-1 cells. **c**, E2F1 depletion reduced expression of *PLACT1* in AsPC-1 cells as detected by qRT-PCR. **d-e**, E2F1 overexpression increased expression of *PLACT1* in PANC-1 (d) and AsPC-1 (e) cells as detected by qRT-PCR. **f**, Luciferase reporter assays showed that E2F1 knockdown reduced the transcriptional activity of PLACT1 promoter in AsPC-1 cells. **g**, Luciferase reporter assays demonstrated that depletion of E1 decreased the transcriptional activity of PLACT1 promoter in AsPC-1 cells. Statistical significance was evaluated using two-tailed *t*-tests and ANOVA followed by Dunnett′s tests for multiple comparisons. Figures with error bars represent standard deviations of three independent experiments. **p* < 0.05 and ***p*< 0.01.
