# Supplementary material for: lncRNA-PLACT1 sustains activation of NF-κB pathway through a positive feedback loop with IκBα/E2F1 axis in pancreatic cancer
Source: Mol Cancer. 2020 Feb 21;19:35. doi: 10.1186/s12943-020-01153-1 (PMC7033942; doi:10.1186/s12943-020-01153-1)
Supplement: Supplementary file 12 — Additional file 12: Table S2. Primer and probes of experiments. [file 12943_2020_1153_MOESM12_ESM.docx]

**Table S2. Primer and probes of experiments.**

| **Gene (human)** | **Sequence (5′ to 3′)** | **Application** |
| --- | --- | --- |
| GAPDH | F: ATCACCATCTTCCAGGAGCGA  R: CCTTCTCCATGGTGGTGAAGAC | qRT-PCR |
| PLACT1 | F: TCTCATCCCGTGTCATGTGCC  R: GTTTCAGGTCTCCATGCCAGTG | qRT-PCR |
| HnRNPA1 | F: TCCATTATAGCCATCCCCACT  R: GAAAAGCCCTGTCAAAGCAAG | qRT-PCR |
| IκBα | F: TGGTCAGTGCCTTTTCTTCAT  R: GGAGTACGAGCAGATGGTCAA | qRT-PCR |
| E2F1 | F: GGACCTTCGTAGCATTGCAGAC  R: TCAGGGCACAGGAAAACATCG | qRT-PCR |
| P65 | F: GAAGAGCAGCGTGGGGACTAC  R: CAAAGATGGGATGAGAAAGGACAGG | qRT-PCR |
| PAI1 | F: ACCGCAACGTGGTTTTCTCA  R: TTGAATCCCATAGCTGCTTGAAT | qRT-PCR |
| IL-1β  TGF-β  TNF-a  IL8  IL6  MDM2  STAT3  IL23A  IGFBP3  c-REL  P50  P52  REL-B  U1 | F: CACGTGGAGCTGTACCAGAA  R: GAACCCGTTGATGTCCACTT  F: TGCCTTCAGCAGAGTGAAGA  R: GTCTTGGTTCTCAGCTTGGG  F: GAGGAAGCAGGTTAATTGGAAGG  R: CCCAGAATCAATGTGAGCTGAG  F: GGGTGGAAAGGTTTGGAGTAT  R: TAGGACAAGAGCCAGGAAGAA  F: ACTCACCTCTTCAGAACGAATTG  R: CCATCTTTGGAAGGTTCAGGTTG  F: GACGTAGAGGCGAGGATTCC  R: GCTGGGAGTGCCGTATGTC  F: CAGCAGCTTGACACACGGTA  R: AAACACCAAAGTGGCATGTGA  F: CTCAGGGACAACAGTCAGTTC  R: ACAGGGCTATCAGGGAGCA  F: AGAGCACAGATACCCAGAACT  R: GGTGATTCAGTGTGTCTTCCATT  F: GCAGAGGGGAATGCGTTTTAG  R: AGAAGGGTATGTTCGGTTGTTG  F: AACAGAGAGGATTTCGTTTCCG  R: TTTGACCTGAGGGTAAGACTTCT  F: ATGGAGAGTTGCTACAACCCA  R: CTGTTCCACGATCACCAGGTA  F: CAGCCTCGTGGGGAAAGAC  R: GCCCAGGTTGTTAAAACTGTGC  F: GGGAGATACCATGATCACGAAGG  R: CCACAAATTATGCAGTCGAGTTTC | qRT-PCR  qRT-PCR  qRT-PCR  qRT-PCR  qRT-PCR  qRT-PCR  qRT-PCR  qRT-PCR  qRT-PCR  qRT-PCR  qRT-PCR  qRT-PCR  qRT-PCR  qRT-PCR |
| PLACT1-3’ RACE-nested | ACGTTGTGGCAGGTTGAACT | 3’ RACE |
| PLACT1-3’ RACE | TGGCTCATGCCTGTAATCTC | 3’ RACE |
| si-PLACT1#1 | F: GGUAAUCAGCCUCCCGAAATT  R: UUUCGGGAGGCUGAUUACCTT | siRNA |
| si-PLACT1#2 | F: GCCAGUGAAAUGUCACCUCTT  R: GAGGUGACAUUUCACUGGCTT | siRNA |
| si-P65#1 | F: CCCUAUCCCUUUACGUCAUTT  R: AUGACGUAAAGGGAUAGGGTT | siRNA |
| si-P65#2  si-E2F1#1  si-E2F1#2  si-hnRNPA1#1  si-hnRNPA1#2  IκBα-ChIP  PLACT1-ChIP  PLACT1-ChIP | F: GAUGAAGACUUCUCCUCCATT  R: UGGAGGAGAAGUCUUCAUCTT  F: GACCACCUGAUGAAUAUCUTT  R: AGAUAUUCAUCAGGUGGUCTT  F: CUUCGGAGAACUUUCAGAUTT  R: AUCUGAAAGUUCUCCGAAGT  CAGCUGAGGAAGCUCUUCATT  Mixed:rCrArArCrUrUrCrGrGrUrCrGr  UrGrGrArGrGrAdTdT  F: ATTCAGTCCATGGCTTGCAG  R: TCCTTGCTCTCTTGTCCTGG  F: GCTGGTGTGGCTGGGAGTT  R: CGATGTATCGGGAAGTTTCGGAAT  F: CTACGCCCAGCCATCATTTC  R: GCAAGTGAGCCTTCTTTCCC  F: TCCTATTCAGCAGTTTCCCCA  R: GGAACTTTTAGCTTCGCCCC  F: ATTCAGTCCATGGCTTGCAG  R: TCCTTGCTCTCTTGTCCTGG  F: TGCAGAACTACAGAGGCCTC  R: AAAGGGGTTTCGGCTCACTA  F: AGCCATGGTCACCCTTACAA  R: TGGGACATCTCAATTCCAGGT | siRNA  siRNA  siRNA  siRNA  siRNA  ChIP-qPCR  ChIRP-qPCR |
| PLACT1#1_odd  PLACT1#2_odd | CCGTGGTTGAGGAACAGAAG  TGGGACCAAGTGGCAAAACG | ChIRP probes |
| PLACT1#3_odd  PLACT1#4_odd  PLACT1#5_odd  PLACT1#1_even  PLACT1#2_even  PLACT1#3_even  PLACT1#4_even  PLACT1#5_even | AACTGGGTCACAGAAAGGCT  CGTAAAAGCCTCTTAGAGGC  CGGTGGAGTTGAGGTGACAT  TTCAAGACTCTTAACAGGTT  ATAGTCTCCCGTGGTTTAAG  AAGTCTCATCCCGTGTCATG  TTGTGGCAGGTTGAACTTTC  GCAAATTTTGTGCATTCACA |  |

Abbreviations：F: forward; R: reverse.
