# Supplementary material for: lncRNA-PLACT1 sustains activation of NF-κB pathway through a positive feedback loop with IκBα/E2F1 axis in pancreatic cancer
Source: Mol Cancer. 2020 Feb 21;19:35. doi: 10.1186/s12943-020-01153-1 (PMC7033942; doi:10.1186/s12943-020-01153-1)
Supplement: Supplementary file 13 — Additional file 13: Table S3. Antibodies of experiments. [file 12943_2020_1153_MOESM13_ESM.docx]

**Table S3. Antibodies of experiments.**

| **Product** | **No. of Catalogue** | **Supplier** |
| --- | --- | --- |
| **Primary antibody:**  ***IHC:***  mouse anti-ki67  ***Western blotting:***  rabbit anti-P65  rabbit anti-p-P65  mouse anti-IκBα  rabbit anti-p-IκBα  rabbit anti-IKKβ  rabbit anti-p-IKKβ  rabbit anti-GAPDH  rabbit anti-E2F1  mouse anti-hnRNPA1  rabbit anti-Lamin B1  mouse-anti-β-Actin  rabbit-anti-EZH2  ***RIP:***  rabbit anti-hnRNPA1  ***ChIP:***  rabbit anti-E2F1  rabbit anti-H3K27me3  ***Immunofluorescence*:**  rabbit anti-P65 | ZM-0166  8242  3033  4814  2859  2678  ab59195  5174  3742  ab5832  13445  3700  [5246](https://www.cst-c.com.cn/products/primary-antibodies/ezh2-d2c9-xp-rabbit-mab/5246?site-search-type=Products)  8443  3742  9733  8242 | Sino, Biological  Cell Signaling Technology  Cell Signaling Technology  Cell Signaling Technology  Cell Signaling Technology  Cell Signaling Technology  Abcam  Cell Signaling Technology  Cell Signaling Technology  Abcam  Cell Signaling Technology  Cell Signaling Technology  Cell Signaling Technology  Cell Signaling Technology  Cell Signaling Technology  Cell Signaling Technology  Cell Signaling Technology |
| **Secondary antibody:**  ***IHC:***  Goat anti-rabbit IgG-HRP  Goat anti-mouse IgG-HRP  ***Western blotting :***  anti-mouse IgG HRP-linked Ab  anti-rabbit IgG HRP-linked Ab  ***Immunofluorescence*:**  goat anti-mouse IgG Alexa 488 | SA00001-15  SA00001-1  7076  7074  A-11029  12-371B  PP64B | Proteintech  Proteintech  Cell Signaling Technology  Cell Signaling Technology  Invitrogen  Millipore  Millipore |
