# Supplementary figures and images for: lncRNA-PLACT1 sustains activation of NF-κB pathway through a positive feedback loop with IκBα/E2F1 axis in pancreatic cancer
Source: Mol Cancer. 2020 Feb 21;19:35. doi: 10.1186/s12943-020-01153-1 (PMC7033942; doi:10.1186/s12943-020-01153-1)

**Figure S9**

**
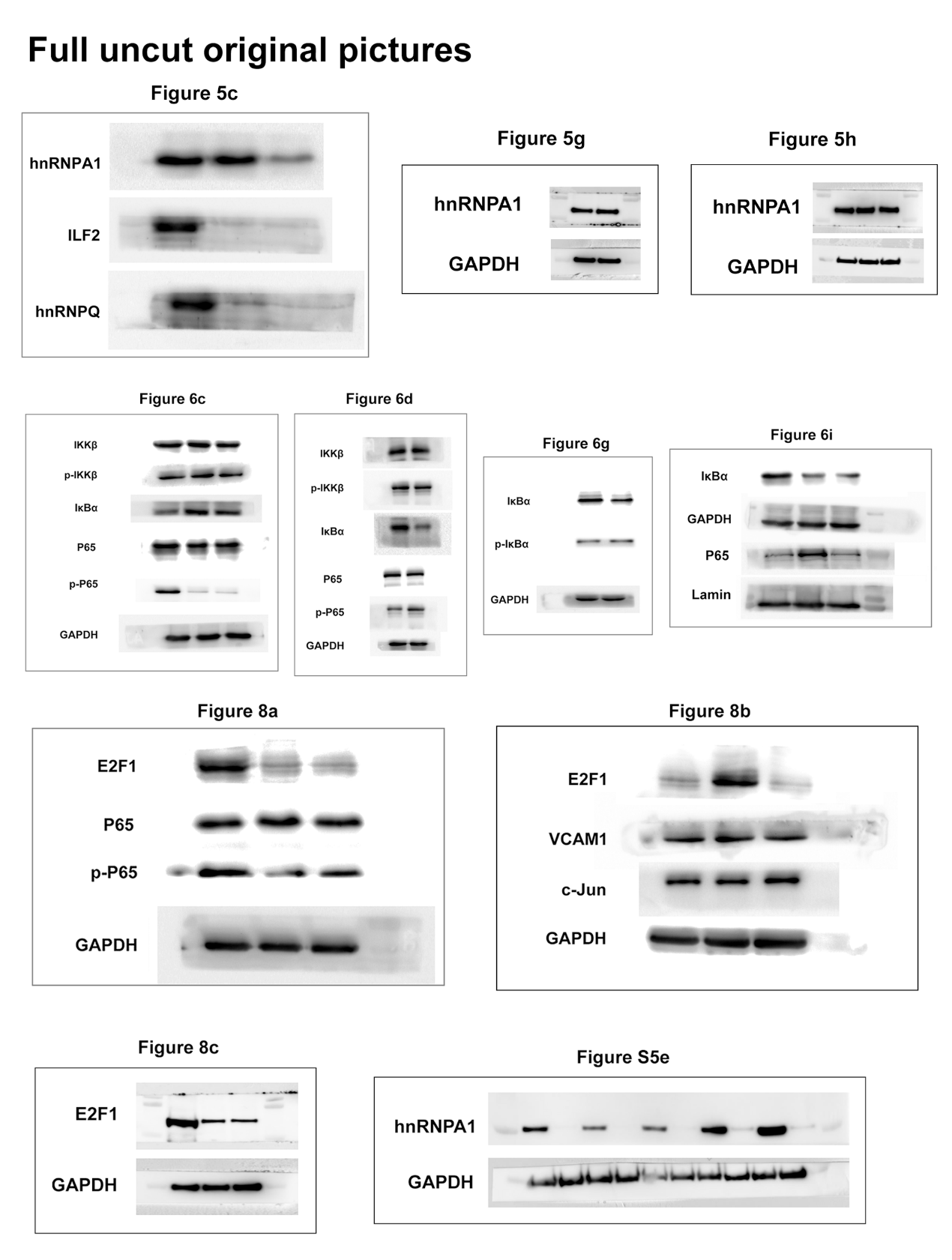
**

**Figure S9. Full uncut original pictures.**

Supplement: Supplementary file 14 — Additional file 14: Figure S9. Full uncut original pictures. [file 12943_2020_1153_MOESM14_ESM.docx]
