# Supplementary material for: lncRNA-PLACT1 sustains activation of NF-κB pathway through a positive feedback loop with IκBα/E2F1 axis in pancreatic cancer
Source: Mol Cancer. 2020 Feb 21;19:35. doi: 10.1186/s12943-020-01153-1 (PMC7033942; doi:10.1186/s12943-020-01153-1)
Supplement: Supplementary file 15 — Additional file 15: Table S4. The possible TFO and TTS predicted for PLACT1 and IκBα promoter. [file 12943_2020_1153_MOESM15_ESM.docx]

**Table S4. The possible TFO and TTS predicted for PLACT1 and IκBα promoter.**

| **Oligo ID** | **TFO (5′-3′)** | **Oligo ID** | **TTS (5′-3′)** | **Score** |
| --- | --- | --- | --- | --- |
| **TFO1** | UCCUGAGCCUCCAUUGUUU | **TTS1** | AGGACCGGCAGGTTGGCAA | 58 |
| **TFO2** | AUCUUUUACUUUCAAU | **TTS2** | TAGGAAGTGATTTGAG | 67 |
| **TFO3** | GAGACUAUCUGGCACA | **TTS3** | CTCTGACCGAAGTTTG | 68 |
| **TFO4** | CAGGGCGACCUUGGGGC | **TTS4** | GTCCCCCAGGACAAGA | 62 |
| **TFO5** | CGUCACUACCGGGCG | **TTS5** | GCAATATGACTTTTA | 44 |

Abbreviation：TFO, Triplex-forming oligos; TTS, triplex target sites; Score, triplex-forming potential score.
